# Evaluation of osteoarthritic features in peripheral joints by ultrasound imaging: A systematic review

**DOI:** 10.1016/j.ocarto.2021.100194

**Published:** 2021-07-16

**Authors:** Prue Molyneux, Catherine Bowen, Richard Ellis, Keith Rome, Mike Frecklington, Matthew Carroll

**Affiliations:** a School of Clinical Sciences, Auckland University of Technology, New Zealand; b Active Living and Rehabilitation: Aotearoa New Zealand, Health and Rehabilitation Research Institute, School of Clinical Sciences, Auckland University of Technology, New Zealand; c School of Health Sciences, Faculty of Environmental and Life Sciences, University of Southampton, UK; d Centre for Sport, Exercise and Osteoarthritis Versus Arthritis, University of Southampton, Southampton, UK

**Keywords:** Osteoarthritis, Ultrasound imaging, Peripheral joints

## Abstract

**Objective:**

To determine how structural and inflammatory osteoarthritis (OA) features in peripheral joints are assessed, defined and graded by ultrasound (US) imaging.

**Design:**

MEDLINE, CINAHL, Cochrane and SPORTDiscus were systematically searched in March 2021. To be eligible, studies needed to (1) include participants with peripheral joint OA, and (2) used grey scale USI or power Doppler (PD) to assess one or more US features in peripheral joints of the hands and feet. Methodological quality of all included studies was assessed using the Critical Appraisal Skills Program (CASP) tool.

**Results:**

A total of 159 citations were identified for screening. Thirty-two articles were included for final analysis and were of good methodological quality. Thirty articles evaluated US features of hand OA and two assessed US OA features in the foot. There were inconsistencies between studies in terms of what US features were assessed, how these features were defined and what grading system was applied to determine degree of osteoarthritic change.

**Conclusion:**

The review found inconsistencies in the definition of synovial pathology. Consequently, it is unclear whether synovial pathology is best represented as separate entities or combined as a single domain, termed “synovitis”. How OA US features were defined and graded has largely been extrapolated from recommendations originally constructed for populations with rheumatoid arthritis (RA). Given the prognostic value of synovitis for OA progression and the reduced degree of inflammation experienced in OA compared to RA, the validity of applying definitions, grading systems and atlases originally developed for inflammatory arthritis needs consideration.

## 1 Introduction

Osteoarthritis (OA) is a global health burden and leading cause of chronic pain, joint stiffness, functional limitation, and disability among older adults [1,2]. OA is a degenerative joint disease and affects multiple structures; including the perichondral and subchondral bone and associated joint capsular structures [[3], [4], [5], [6]]. Our knowledge of foot and hand OA substantially lags behind that of other joint sites, such as the knee and hip [[7], [8], [9], [10]], for which the research evidence is more advanced. However, foot and hand OA are also important contributors to the burden of OA and have a significant negative impact on physical mobility and health-related quality of life [[11], [12], [13]].

Plain radiography represents the gold standard imaging modality for the visualisation of bony change and the diagnosis of radiographic OA [14,15]. Although radiographic imaging can detect joint space narrowing and bony alterations [16], it has numerous shortcomings in diagnosing OA. At the point where structural damage is evident radiographically, joint structure and function may be significantly impaired. Once the joint has reached this point, patient outcomes and management are limited. Radiographic imaging cannot directly visualise articular cartilage or detail the soft tissue changes in and around joints [17]. Radiographic findings are also poorly associated with clinical symptoms [18,19].

Significant advances have been made in the field of imaging, allowing a more accurate evaluation of both bone and soft-tissue abnormalities [20]. Ultrasound (US) imaging presents an alternative to plain radiography in the diagnosis of OA due to its ability to detect features present during disease progression, related both to inflammation and structural damage [5,[21], [22], [23], [24]]. US has proved to be a reliable and valid imaging technique to assess OA features when compared with MRI [25,26]. US can be readily used chairside, presents a lower cost, is widely available, is not contraindicated for some patients, and does not require intravenous contrast for assessment of active synovitis. US has been shown to have high sensitivity to detect subclinical (absence of clinical symptoms) inflammatory joint pathology [3,27] and provides excellent resolution of superficial tissues/structures [26,28,29]. Given the ability of US to depict tissue-specific morphological changes before the onset of pain and before the point of irreversible structural damage, US imaging may play a fundamental role in the earlier detection and assessment of peripheral joint OA [30]. Earlier detection would provide the capacity to alter the progression of the disease and improve quality of life.

The application of US imaging has enhanced the understanding of the complex, multi-tissue processes underpinning the OA phenotype [31]. However, the role of US for OA diagnosis in peripheral joints has not been clearly defined. To further understand this role, the aims of this study were to critically evaluate and summarise relevant studies that have used US to evaluate OA features in peripheral joints of the hands and feet. The primary questions investigated in the review were: What US features are associated with OA in peripheral joints? How are US features in peripheral joints defined and graded? What is the reliability of grading the US features?

## 2 Methods

### 2.1 Search strategy

**Fig. 1:**
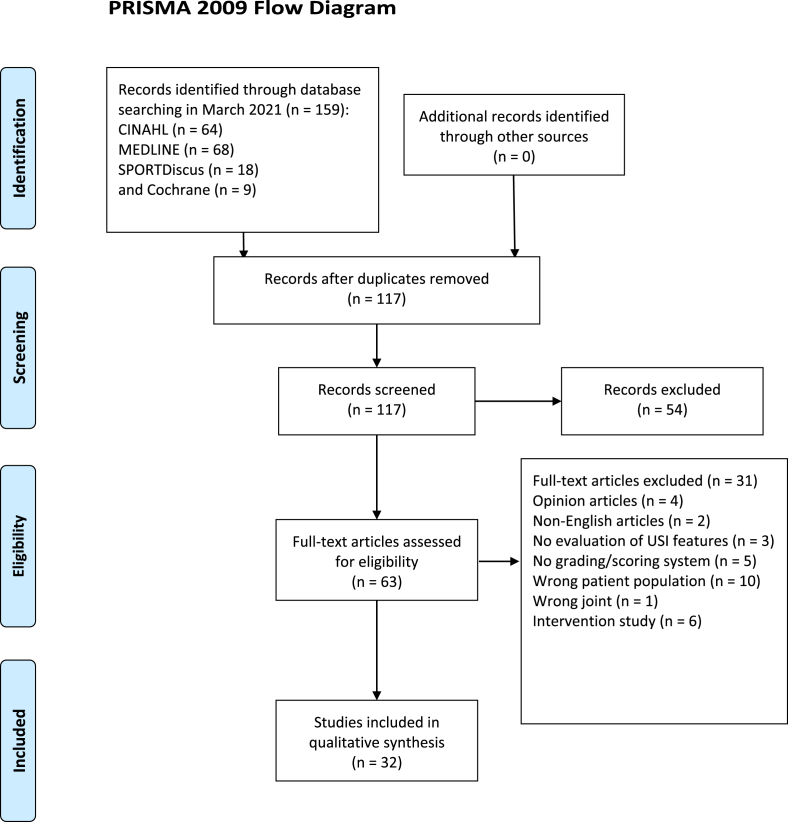
PRISMA guidelines.

This systematic review is reported in accordance with the Preferred Reporting Items for Systematic Reviews and Meta-Analyses (PRISMA) guidelines [32] (Fig. 1). The identification of articles for the systematic review was completed with a comprehensive search of titles and abstracts from key electronic databases and additional records (Table 1). The search was conducted between July 2020 and March 2021. The electronic databases MEDLINE, CINAHL, Cochrane and SPORTDiscus were systematically searched from their earliest record (1997) to 2021. Broad-ranging search terms were agreed on by the authors (PM and MC). A secondary search was performed to address publication bias by searching the Open Grey literature (to search for unpublished literature and ongoing trials) and Google Scholar [33]. All titles and abstracts identified from the search were downloaded into EndNote version ×9 (Thomson Reuters, Philadelphia, PA USA).

**Table 1:** Search strategy.

MEDLINE, CINAHL, Cochrane and SPORTDiscus		
1	Subject term	Osteoarthritis
2	Keywords	Foot or feet or hand
3	Subject term	Ultrasonography
4	Keywords	Ultrasonograph∗ or Sonograph∗ or Ultrasound or US or MSUS or Doppler or power Doppler or PDUS or Colour Doppler or Elastograph∗
5	Keywords	Features or Characteristics or Osteophyte or Synovitis or Cartilage or Effusion or synovial hypertrophy or Erosion or Vascularisation or Neovascularisation
6	Keywords	Atlas or Grad∗ or Scor∗or severity or assess∗ or evaluat∗
7	Combine	1 and 2
8	Combine	3 and 4
9	Combine	5 and 6
10	Combine	7 and 8 and 9

### 2.2 Inclusion and exclusion criteria

The studies were cross-referenced with duplicates removed. The retrieved articles were imported into Covidence systematic review software, Veritas Health Innovation, Melbourne, Australia [34]. In the first stage of selection the titles and abstracts were independently screened by PM and MC. Subsequently, the full texts of the selected articles were retrieved and judged against the inclusion and exclusion criteria (Table 2). The selected studies were discussed between authors until consensus for inclusion was achieved. In cases of non-consensus, a third author's opinion was planned for consultation; however, this was not required. The eligibility criteria were initially applied to all titles and abstracts, and later to full-text articles if more detail was required. All studies that met the inclusion, had their reference lists hand searched for further included articles. When the included studies referred to a previous paper for methodology or reliability, that paper was accessed, and appraised for inclusion against the selection criteria. This systematic review was registered with the international database of prospectively registered systematic reviews in health and social care (PROSPERO), registration number CRD42021199396.

**Table 2:** Eligibility criteria.

Inclusion criteria:
• participants were over 18 years old
• participants (cases) with osteoarthritis, defined by either radiographically confirmed osteoarthritis, patient reported osteoarthritis, or clinical diagnosis
• they used grey scale ultrasound imaging or power Doppler to assess one or more ultrasound imaging features in peripheral joints of the hand and feet
Exclusion criteria:
• were unpublished; non-peer-reviewed; do not involve humans; are in vitro studies; opinion articles; letters to the editor; non-English articles and abstracts
• included participants with inflammatory arthritis or a neurological, endocrine or metabolic disorder.
• only evaluated ultrasound imaging features evaluated in other joints, aside from those of the hands and feet
• studies that utilised ultrasonography only for guiding injections and did not report any USI feature data or findings of the ultrasonography examination

### 2.3 Methodological quality assessment

The methodological quality of included articles was appraised independently by two reviewers (PM, MF) using the Critical Appraisal Skills Program (CASP) case control and cohort checklists [35]. The CASP tools are succinct and effectively cover the areas needed for critical appraisal of evidence [36]. The cohort and case control checklists comprise a series of 12 and 11 questions, respectively. Completion of the checklists provides a systematic and comprehensive way of appraising studies to determine whether their findings are valid, accurate and meaningful at a local level. Each criterion was weighted by applying a three-point scale; No ​= ​criterion not met (0 points); Yes ​= ​criterion totally met (1 point); Can't tell ​= ​criterion partially met (C/T). A total score was generated out of 14 points for cohort studies and a total of 12 points for case control studies. A consensus meeting was held to resolve any disagreement between the reviewers. Following methodological assessment, articles were grouped and discussed according to US feature, definition, and applied grading system.

### 2.4 Data extraction

The following information was extracted from all included studies: study characteristics; author's name, year of publication, study design and aim(s), and outcome measure(s) reported. Participant characteristics including sample size, gender, mean age (years), mean BMI (kg.m^−2^), and symptom duration were also extracted (Supplementary data 1). Additionally, the following US measurement techniques were extracted: what OA features were imaged, how the US features were graded (dichotomous or on a semiquantitative scale), if an US atlas was used, the sonographer(s) involved in the assessment, and all reliability data that were recorded (Supplementary data 2).

## 3 Results

### 3.1 Selection and characteristics of studies

A total of 159 citations were identified for screening with 32 articles included for final analysis. Thirty articles evaluated US features of hand OA [24,25,[37], [38], [39], [40], [41], [42], [43], [44], [45], [46], [47], [48], [49], [50], [51], [52], [53], [54], [55], [56], [57], [58], [59], [60], [61], [62], [63], [64]] and two assessed US OA features in the foot [65,66]. Twenty-seven hand OA studies assessed the proximal and distal interphalangeal joints [24,25,37,38,[41], [42], [43], [44], [45], [46], [47], [48], [49], [50], [51], [52], [53], [54], [55], [56], [57],[59], [60], [61], [62], [63], [64]], 22 studies assessed the metacarpophalangeal and carpometacarpal joints [24,25,[38], [39], [40], [41], [42], [43],^45^,[48], [49], [50],^52^,^53^,[56], [57], [58], [59], [60], [61], [62], [63]], and four studies assessed the first interphalangeal joint [43,[48], [49], [50]]. With regards to the two-foot studies, both assessed US imaging OA features in the first metatarsophalangeal joint (MTPJ) and the midfoot [65,66]. One study evaluated and graded the midfoot joints as a single joint complex [65] and one evaluated and graded each joint at the midfoot and forefoot level [66].

A total of 3069 participants were reported (654 male, 2330 female) of which 2952 were diagnosed with peripheral joint OA. Sex was not reported in two studies, involving eighty-five participants [43,60]. The mean age of participants ranged from 51.1 to 74.5 years old. Mean BMI was reported in 16 studies and ranged from 24.9 ​kg/m^2^ to 28.4 ​kg/m^2^. Eleven studies reported disease duration (range, 3.2–18.5 years). Ethnicity of the study population was reported by one study [44]. Five studies delineated separate OA sub-groups as erosive and non-erosive hand OA [46,51,55,56,59]. All included studies were observational studies published after 2008, 26 were cohort and six were case-control studies. The aims, participant characteristics and how peripheral joint OA was defined of all included studies are presented in Supplementary data 1. Meta-analyses were not deemed appropriate based on the variation in features imaged, specific joints that were imaged and how US features were defined and graded.

### 3.2 Quality assessment of studies

The quality scores for the included cohort studies ranged from 4 to 14/14 on the CASP quality checklist. The quality scores for the included case control studies ranged from 5 to 8/12 on the CASP quality checklist. The quality of all included studies was summarised in a table format (Supplementary data 3). Due to the exclusion of intervention studies, questions related to treatment effect were not applicable.

### 3.3 US features associated with OA

There was wide variation across all studies in relation to what US imaging features were assessed. The following inflammatory US features were investigated by the included studies: synovitis, synovial hypertrophy, joint effusion, tenosynovitis, and power Doppler (PD) signal. PD signal was the most reported US feature across all studies (n ​= ​24) [24,[37], [38], [39], [40], [41],^44^,[46], [47], [48], [49], [50], [51],[53], [54], [55],^57^,^59^,^60^,[62], [63], [64], [65], [66]]. There were also inconsistencies between the different entities of synovial pathology, making comparison between studies difficult. Of the 32 studies included in the review, 16 assessed synovitis [24,[37], [38], [39],^41^,^42^,^46^,^49^,^54^,^56^,^57^,^59^,^60^,[62], [63], [64]], of which eight studies combined joint effusion and synovial hypertrophy as a single domain, termed “synovitis” [24,39,49,52,54,59,62,63], whereas five studies assessed synovitis and joint effusion as separate entities [41,49,56,57,64]. Furthermore, several studies clearly differentiated between synovial hypertrophy and joint effusion; with 14 studies having assessed joint effusion [40,41,[47], [48], [49], [50], [51],^53^,[55], [56], [57],^65^,^66^] and 12 studies assessed synovial hypertrophy [40,41,44,[47], [48], [49], [50], [51],^53^,^55^,[64], [65], [66]] as separate entities (US features). Tenosynovitis was reported by two studies [56,57]. OA features indicative of structural damage included osteophytes, which were reported in 18 studies [25,38,39,41,43,45,50,52,[54], [55], [56], [57],[60], [61], [62], [63],^65^,^66^] joint erosions, reported in seven studies [38,39,41,51,54,56,57], cartilage breakdown, reported in five studies [45,54,58,59,65], and joint space narrowing reported in three studies [54,61,62].

### 3.4 Defining US features associated with OA

Definitions of US features for all included studies are presented in Table 3. There was no consistent use of US definitions used to define each US feature associated with OA. Common inconsistencies were evident between individual studies interpretation of the different entities of synovial pathology. How individual studies differentiated synovitis, joint effusion and synovial hypertrophy as either a single or combined entity determined how that feature was defined. Definitions of the imaging appearance of the US features were provided in 23 studies [25,38,41,45,46,49,[51], [52], [53], [54], [55], [56], [57], [58], [59], [60], [61], [62],[64], [65], [66], [67], [68]]. Only 16 of those studies included a definition for each US feature evaluated, of which four studies referred to a previous study for definition of pathology [38,41,49,51] (Supplementary data 2). Eight studies did not define any of the US features evaluated [37,40,[42], [43], [44],^47^,^48^,^50^].

**Table 3:** Defining and of grading of OA features using USI.

Sonographic feature	Definition	Grading system		
		Semiquantitative	Dichotomous (present or absent)	Continuous (mm)
Osteophytes	Cortical protrusion >1 ​mm at the end of the normal bone contour or at the margin of the joint	[38,42,44,49,54,55,60,63,65]	[37,40,51,53,56,57,60,62]	NR
	[38,44,51,53,[55], [56], [57],[60], [61], [62],^66^]			
Erosions	Irregularity of the hyperechoic cortical bone, evident in two perpendicular planes	NR	[37,40,56,57]	NR
	[38,40,50,57]			
Articular cartilage damage	Loss of the normal sharpness of cartilage interfaces, irregularities/thinning of the margins and/or increased echogenicity of the cartilage	[44]	[53,58,65]	[59]
	[53,58,65]			
Synovitis	Distension of the joint capsule ≥1.5 ​mm in its anteroposterior diameter with compressible material	[24,[36], [37], [38],^40^,^45^,^48^,^60^,^61^,^63^]	[41,53,56,57,59,60]	NR
	[45,53]			
	A composite of effusion and synovial thickening			
	[24,38,48,[59], [60], [61]]			
Synovial hypertrophy	Abnormal hypoechoic intraarticular tissue or thickening of the synovial membrane that is non-displaceable and poorly compressible and is often associated with increased vascularity	[39,43,[46], [47], [48], [49],^52^,^54^]	[40,50,65,66]	NR
	[38,48,52]			
Joint effusion	Abnormal hypoechoic or anechoic intra-articular material that is displaceable and compressible but does not exhibit power Doppler signal	[24,39,[47], [48], [49],^52^,^54^,^64^]	[40,50,56,57,65,66]	NR
	[38,48,50,54,56,57,64,66]			
Power Doppler activity	A pulsating colour spot found within the synovial structure, which represented presence of vascularisation	[24,36,37,39,40,[43], [44], [45],[47], [48], [49],^52^,^54^,[61], [62], [63], [64], [65]]	[36,38,50,53,57,59,66]	NR
	[24,38,40,45,48,50,52,53,61,62,64]			
Joint space narrowing	Decrease in the space between the cortical margins	NR	[53,61,62]	NR
	[53,61,62]			
Tenosynovitis	A hypoechoic rim around tendon with or without PD signal	NR	[56,57]	NR
	[56,57].			

NR, Not reported.

### 3.5 Grading US features associated with OA

A summary of how each US feature was graded in the 32 reviewed studies is presented in Table 3. There was also no consistent way in which each US feature was graded to classify the degree of pathological change in joint tissue. The variation between studies made comparison difficult and leaves grading of US features open to interpretation. The grading systems applied were either dichotomous, semiquantitative, or continuous. The majority of studies applied a previously developed grading system to evaluate each US OA feature (Table 4). Table 4 outlines studies that cited a previously developed grading system.

**Table 4:** Origin of grading system applied to evaluate USI OA features.

Sonographic feature	Origin of grading system (number of studies which applied a grading system)				
	Semiquantitative		Dichotomous (present or absent)		Continuous (mm)
	Grading system referenced	Studies that referenced system	Grading system referenced	Studies that referenced system	
Osteophytes	4x Mathiessen [27]	[38,42,44,65]	1x Keen [60]	[62]	NR
	2x Keen [60]	[27,63]			
	1x Kortekaas [48]	[49]			
	1x Hammer [69]	[27]			
Erosions	NR	NR	1x Wakefield [80]	[40]	NR
Articular cartilage damage	NR	NR	1x Iagnocco [58]	[65]	NR
Synovitis	5x Keen [60]	[36,38,48,61,63]	1x Hammer [69] 1x Keen [60] 1x Wakefield [80]	[41] [59] [60]	NR
	2x Hammer [69]	[45,68]			
	1x Terslev^88^	[37]			
	1x Mandl^89^	[40]			
	1x Mathiessen [27]	[75]			
Synovial hypertrophy	5x Kortekaas [48]	[39,46,47,49,52] [43,54] [38,48] [65] [40]	1x Terslev^88^	[65]	NR
	2x Szkudlarek^90^				
	2x Keen (2008) [60]				
	1x D'Agostino^91^				
	1x Mandl^89^		1x Wakefield [80]	[50]	
Joint effusion	5x Kortekaas [48]	[39,46,47,49,52] [54,64] [40] [48]	1x Terslev^88^	[65]	NR
	2x Szkudlarek^90^				
	1x Mandl^89^				
	1x Keen [60]		1x Wakefield [80]	[50]	
Power Doppler activity	5x Kortekaas [48]	[39,46,47,49,52] [36,48,61,63] [42,45] [37] [40] [43] [65]	2x Wakefield [80]	[38,50]	NR
	4x Keen [60]				
	2x Hammer [69]1x Terslev^88^				
	1x Mandl^89^				
	1x Szkudlarek^90^				
	1x D'Agostino^91^		1x Keen [60]	[62]	
Joint space narrowing	NR	NR	NR	NR	NR
Tenosynovitis	NR	NR	NR	NR	NR

NR, Not reported.

### 3.6 Use of an US atlas

Of the 32 studies included, six reported using an US atlas to assist with grading of US features [25,39,42,45,46,68]. Across the six studies, six different US features were assessed. An US atlas was only used as a reference to evaluate synovitis, PD activity, cartilage damage and osteophytes. Three studies [24,42,46], applied the same US atlas that was originally developed to assess synovitis in rheumatoid arthritis (RA) [69]. One study developed an original US atlas to grade osteophytes in finger joints [25], which was later used by two studies to grade severity of finger joint osteophytes [39,45]. The later study also developed a new US atlas to grade cartilage [45]. Hammer et al. [45] was the only included study that used multiple US atlases to assist grading of all features evaluated. Neither foot study used an US atlas to assist grading.

### 3.7 Reliability of grading US features

Twenty studies evaluated reliability of grading US features [25,37,39,44,45,[48], [49], [50], [51], [52],[55], [56], [57], [58], [59], [60], [61], [62],^65^,^68^]. The reliability of grading PD signal and osteophytes were the most commonly evaluated features. Seventeen studies assessed intra-rater reliability of grading US features [25,39,44,45,[48], [49], [50], [51],^55^,[57], [58], [59], [60], [61], [62],^65^,^68^] and nine assessed inter-rater reliability of grading US features [25,37,44,45,52,58,60,65,68]. Data was predominantly assessed by Kappa statistics and four studies assessed data by intra-class correlation coefficients (ICC) [[48], [49], [50],^55^]. One study assessed agreement between US and magnetic resonance imaging (MRI) [56]. Supplementary data 4 outlines which studies assessed reliability for each US feature, which grading system was applied to each feature, what type of reliability was evaluated, and the sonographer(s) involved in the assessment.

## 4 Discussion

This review investigated what US features were associated with OA in peripheral joints, how these features were defined and graded, and the reliability of assessing US features. There were inconsistencies between studies in terms of what US features were assessed (synovitis, synovial hypertrophy, joint effusion, tenosynovitis, PD signal, osteophytes, joint erosions, cartilage breakdown, and joint space narrowing), how these features were defined and what type of grading system (dichotomous, semiquantitative, or continuous) was applied to determine degree of osteoarthritic change. The methodological quality of the included studies as assed by the CASP tool, demonstrated that only three of the 32 studies met all the checklist criteria, with eight studies scored at 50% or less of the criteria.

OA is characterised by both structural damage and inflammatory abnormalities [4,66]. US enables evaluation of articular cartilage, bone, and soft tissue [20,[70], [71], [72], [73]]. The traditional view of OA as a cartilage-only disease is obsolete and attention has now turned to the prognostic value and role of synovitis [74]. Several studies have demonstrated an association between active synovitis and structural OA progression [24,47,48,51]. This association indicates that US could identify those patients, or those joints at greatest risk for progression and provide capacity for earlier detection and assessment of OA-related change in peripheral joints. Mathiessen et al. [75] highlighted the importance of US to obtain an early diagnosis showing that US could detect inflammatory changes five years earlier than what could be seen radiographically. Kortekaas et al. [48] presented similar findings in hand OA, where osteophytes and joint space narrowing progression were often preceded by PD activity and synovitis. The synovial inflammation exhibited in early OA suggests a window of opportunity may exist for interventions targeting the inflammatory processes [76], thus providing the ability to intervene before irreversible structural damage occurs [[77], [78], [79]]. However, the use of US to categorise OA-based change is limited by inconsistencies and the lack of consensus as to which US features should specifically be evaluated to diagnose and grade peripheral joint OA.

Defining US features also remains inconsistent as there are no universally accepted definitions for US features in OA. The OMERACT ultrasound working group have recommended provisional definitions of US features considered to represent inflammatory arthritis [80]. Despite the fact that OA is considered a non-inflammatory disorder, as the leukocyte count is below the threshold that defines inflammatory disorders [81], OMERACT ultrasound definitions were applied to OA in some studies [25,39,41,51,55,59,60,64,66], but not consistently. In terms of defining OA US features the key inconsistency identified in the review was between the different entities of synovial pathology indicative of inflammation. There were discrepancies across studies in terms of how synovitis, synovial hypertrophy and joint effusion were defined and categorised as US features. Consequently, it is unclear whether synovial pathology is best represented as separate entities (joint effusion and synovial hypertrophy) or combined as a single domain, termed “synovitis”. The OMERACT ultrasound group recently proposed a new definition of synovitis detected by US, which encompasses the whole concept of synovitis, “presence of a hypoechoic synovial hypertrophy regardless of the presence of effusion or any grade of Doppler signal” [82]. Due to the recent publication of this study, none of the studies included in this review applied the revised OMERACT definition.

No study reported following an international consensus-based standard for grading OA features. There was no clear consensus as to which type of grading system (dichotomous or semiquantitative) should be applied for specific US features of peripheral joint OA. While dichotomous scoring may be viewed as a simpler method to distinguish between the absence or presence of a feature, it presents no mechanism to determine the progression of peripheral joint OA. Alternatively, semiquantitative systems do enable quantification of disease progression and provide further insight into the degree of osteoarthritic change. However, semiquantitative grading systems applied to OA were adopted from those originally designed and validated to quantify inflammatory change in RA. This assumes that inflammatory pathology is only quantitatively but not qualitatively different between RA and OA [67,83]. Issues related to the subjectivity of semiquantitative systems have also been highlighted, with studies reporting challenges in interpretation and differentiation between grades [37]. In particular, the low frequency of inflammatory pathology that is graded as severe on a semiquantitative system, may be reflective of the reduced degree of inflammation experienced in OA compared to RA [37,48]. This reinforces the need for OA-specific grading systems that truly depict the disease progression of peripheral joint OA.

An US atlas permits the sonographer to have a direct comparison between the detected US features and examples of defined graded images in the atlas, reducing the degree of subjectivity related to grading [45]. Previously published studies have emphasised the need for the development of an US atlas to accompany protocols [84], due to variability in image interpretation [67,85]. This review demonstrated that the use of a US atlas to aid grading of US features in peripheral joints was limited. Significantly, an US atlas which depicts and quantifies the degree of structural and inflammatory change for multiple peripheral joint OA features has not been developed. The review also found that atlas use is limited by two factors. First, despite most studies assessing multiple US features, no study included an atlas that graded more than one US feature. Second, US atlases used to grade OA have been extrapolated from atlases originally developed to grade US features in RA.

The variation in intra-rater and inter-rater reliability from poor to excellent across all studies is attributable to several factors including what US features were evaluated, variation in how each US feature was defined, variation in the type of grading system applied, whether an US atlas was utilised, the use of multiple sonographers involved in the assessment, and the academic background and/or experience of the sonographers. There is a general opinion that US is heavily operator dependent for image acquisition and interpretation [86]. However, US has previously demonstrated a strong correlation with MRI in principal OA features [67]. US has been shown to be as reliable as other imaging modalities when a standardised US acquisition protocol and grading systems is used [86].

This systematic review is not without limitations. Potential sources of heterogeneity include differences in diagnostic criteria, populations, and case definitions, this variation limited the ability to perform meta-analysis. All relevant studies were included in this systematic review, regardless of methodological quality. We restricted the search to studies published in English. Inclusion of data from non-English language studies may alter the outcomes. We excluded studies that included participants with inflammatory arthritis even as a comparator group. Inclusion of participants with RA as a comparator group may have provided more insight or enabled a stronger comparison between grades of inflammation and allowed the direct comparison between definitions and grading systems applied.

Future US imaging studies of peripheral joints will be improved by including more ethnic and age diverse populations, and assessment of changes in asymptomatic healthy controls as well as those who are symptomatic or have radiographic change. The prevalence and burden of OA is not uniform across demographic groups. However, there is a dearth of research examining ethnic differences in peripheral joint OA. Minority populations, especially African American, Hispanic, Māori and Pasifika experience poorer health outcomes (such as pain and disability). Future research should proactively recruit an ethnic diverse population to ensure there is adequate data to undertake an ethnic specific analysis and examine what factors are contributing to these disparities. Future studies should include 3D US to provide further diagnostic information and allow quantification of osteoarthritic change. 3D US provides numerous advantages including visualisation of the coronal plane, image reconstruction, reduced scanning time and limits the influence the sonographers experience has on image acquisition. This would be of particular interest for the determination of the extent of peripheral joint synovitis. Standardisation is also required regarding imaging acquisition protocols, definitions, grading systems, and US atlases. These items align with the recently developed EULAR US recommendation checklist to ensure transparent and comprehensive reporting of US research in rheumatic and musculoskeletal diseases [87]. Addressing these inconsistencies in US research will considerably improve the interpretability, reproducibility and generalisability of the study results [87]. US holds significant promise as a diagnostic tool in OA, providing prognostic information as well as advancing clinical decision making to reduce the burden of peripheral joint OA. As indicated by the review there is a dearth of US research related to foot OA, consequently more foot specific US research is required to understand the progression of foot OA.

## 5 Conclusion

US presents an alternative to plain radiography for the imaging-based diagnosis of peripheral joint OA. However, no standardised US grading system exists to classify and grade the disease process. This review has demonstrated the large degree of variation in what OA features were assessed, how features were defined, and what graded system was applied. The key inconsistency identified was between the different entities of synovial pathology indicative of inflammation. Consequently, it is unclear whether synovial pathology is best represented as separate entities or combined as a single domain, termed “synovitis”. How OA features were defined and graded has largely been extrapolated from recommendations originally constructed for populations with RA. Given the prognostic value of synovitis for OA progression and the reduced degree of inflammation experienced in OA compared to RA, the validity of applying definitions, grading systems and atlases originally developed for inflammatory arthritis needs consideration. This review strengthens the case for further refinement and validation of OA definitions, grading systems and US atlases specific to peripheral joints.

## Contributions

All authors (PM, CB, RF, MF, KR and MC) were responsible for the conception and design of the research. PM and MC were responsible for reviewing articles, analysing data, interpreting the results. PM and MF participated in the Quality Scoring process. All authors were responsible for the preparation and review of the manuscript prior to submission for publication. All authors read and approved the final manuscript.

### Role of the funding source

The project is funded by the 10.13039/501100001505Health Research Council of New Zealand. This organisation had no role in the study design, collection, analysis, or interpretation of the data or in the decision to submit the article for publication.

## Declaration of competing interest

All authors declare they have no competing interests.
