# Supplementary material for: Evaluation of osteoarthritic features in peripheral joints by ultrasound imaging: A systematic review
Source: Osteoarthr Cartil Open. 2021 Jul 16;3(3):100194. doi: 10.1016/j.ocarto.2021.100194 (PMC9718269; doi:10.1016/j.ocarto.2021.100194)
Supplement: Multimedia component 1 [file mmc1.docx]

**Evaluation of osteoarthritic features in peripheral joints by ultrasound imaging: a systematic review**

**Supplementary Material 1: Study design and participant demographics**

| **Foot OA** | | | |  |
| --- | --- | --- | --- | --- |
| **Author** | **Study aim** | **Study design/participant/sample size** | **Definition of OA cohort (inclusion criteria)** | |
| Zabotti ^1^ | Evaluate the level of agreement on US lesions among highly experienced sonographers as well as the intraobserver and interobserver reliability of inflammatory and structural US lesions in patients with foot OA | Delphi and cross-sectional reliability study design. Participants mean (SD) age 67.8 (NSD) years, *n* = 12 (M:F = 2:10) | - Reported foot pain on weight bearing - diagnosis of foot OA based on clinical examination - radiographic criteria based on the LaTrobe Foot Atlas ^2^ | |
| Iagnocco ^3^ | To investigate the prevalence of US abnormalities in the feet of patients with OA and to compare them with clinical findings | Cross-sectional study design. Participants mean (SD) age 65.4 (10.8) years, disease duration (SD) years 1.1 (0.9), *n* = 100 (M:F = 43:57) | Clinical and radiographic signs of OA involving the feet | |

| **Hand OA** | | | |  |
| --- | --- | --- | --- | --- |
| **Author** | **Study aim** | **Study design/participant/sample size** | **Definition of OA cohort (inclusion criteria)** | |
| Fjellstad ^4^ | Explore whether US-detected gray-scale synovitis and PD activity in the interphalangeal and first carpometacarpal joints are associated with pain and physical function in patients with hand OA | Cross-sectional study design. Data from the baseline examination of the Nor-Hand study.  Participants mean (SD) age 60.9 (NSD) years, BMI 25.5 (NSD) kgm^2^, *n* = 290 (M:F = 34:256) | Clinical examination or presence of osteophytes determined by US | |
| Steen ^5^ | Examine the association of structural and inflammatory features of hand OA with local pressure pain thresholds in the Nor-Hand  Study | Cross-sectional study design. Participants mean (SD) age 61 (NSD) years, disease duration (SD) years 6 (NSD), BMI 26 (5) kgm^2^, *n* = 285 (M:F = 34:251) | Evidence of hand OA by ultrasound and/or clinical examination performed by a rheumatologist | |
| Besselink  ^6^ | Assess performance of optical spectral transmission in assessing synovitis in hand and wrist OA. Then compare optical spectral transmission levels between joints with and without US synovitis | Cross-sectional study design. Participants mean (SD) age 64.5 (9.9) years, *n* = (M:F = 5:42) | Recruited from rheumatology clinic. Participants were eligible if they had at least one swollen finger or wrist joint | |
| Oo ^7^ | Determine the associations of OA US features with the extent of pain, function, strength and radiographic scores in symptomatic thumb-base OA | Cross-sectional study design. Participants mean (SD) age 67.0 (7.0) years, disease duration 3.1 (1.1) years, BMI 29.4 (6.7) kgm^2^, *n* = 93 (M:F = 73:20) | - Thumb-base pain at least half of the days in the past month - Average pain ≥40 on a 100mm VAS - Functional Index for HOA - scores ≥6 (Dreiser, Maheu, Guillou, Caspard, & Grouin, 1995) - KL grade ≥2 in the index thumb-base joint | |

| **Author** | **Study aim** | **Study design/participant/sample size** | **Definition of OA cohort (inclusion criteria)** |
| --- | --- | --- | --- |
| Kroon ^8^ | Investigate the associations between US and MRI inflammatory features, structural damage and pain in the thumb base of hand OA patients | Cross-sectional study design. Participants mean (SD) age 60.3 (8.8) years, BMI 27.2 (4.5) kgm^2^, *n* = 87 (M:F = 16:71) | ACR criteria for hand OA ^9^ |
| Sivakumaran ^10^ | Investigate the usefulness of a standardised US examination protocol for hand joints in  diagnosing OA | Cross-sectional study design. Participants mean (SD) age 51.1 (15.3) years, disease duration 4 (NSD) years, *n* = 62 (M:F = 12:50) | Hand OA based on EULAR recommendations ^11^ |
| Magnusson  ^12^ | To explore whether smoking and alcohol use are associated with hand OA features in two  different OA cohorts | Cohort study design.  Radiographic hand OA (MUST cohort)  age 65.3 (8.0) years, BMI 28.4 (4.8) kgm^2^, *n* = 530 (M:F = 155:375)  Oslo hand OA cohort  age 61.6 (5.7) years, BMI 25.6 (4.1) kgm^2^, *n* = 187 (M:F = 17:170) | **MUST cohort** (selected from the general population with self-reported or physician-based or X-ray-diagnosed OA)  radiographic hand OA (KL grade ≥2)  57.4% of these fulfilled the ACR criteria for hand OA  **Oslo hand OA cohort** (consists of people visiting specialist care for their hand OA)  92.5% had radiographic hand OA (KL grade ≥2)  83.4% fulfilled the ACR criteria for hand OA |
| Mathiessen  ^13^ | Determine whether US detected osteophytes (in radiographically and clinically normal finger joints) predicted the development of radiographic and clinical hand OA five years later | Prospective cohort study design.  *N* = 78  From the Oslo Hand OA cohort | ACR criteria for hand OA ^9^ |
| Spolidoro, ^14^ | To assess the correlation between inflammatory sonographic findings and clinical and functional assessments in hand OA and correlate the intraobserver and interobserver reliability | Cross-sectional study design. Participants mean (SD) age 60.7 (8.2) years, disease duration 5.0 (3.6) years, *n* = 60.7 (8.2) (M:F = 2:58) | - ACR criteria for hand OA ^9^ - Older than 40 years - VAS pain score (0-10cm) |
| Hammer  ^15^ | Explore the reliability of highly experienced sonographers in performing semiquantitative  US scoring of cartilage pathology and osteophytes in the finger joints of patients with hand OA | Cross-sectional study design. Participants mean (SD) age 74.5 (NSD) years, BMI 23.2 (3.1) kgm^2^, *n* = 10 (M:F = 0:10) | ACR criteria for hand OA ^9^ |

| **Author** | **Study aim** | **Study design/participant/sample size** | **Definition of OA cohort (inclusion criteria)** |
| --- | --- | --- | --- |
| Haugen ^16^ | To compare the prevalence of synovitis, pain and radiographic progression in non-erosive and erosive hand OA and to explore whether the different rate of disease progression is explained by different levels of synovitis and structural damage | Prospective cohort study design. Participants of the Oslo hand OA cohort  **Non-erosive OA**  Participants mean (SD) age 68.3 (4.6) years, disease duration 16 (NSD) years, BMI 26.5 (3.9) kgm^2^, *n* = 31 (M:F = 4:27)  **Erosive OA**  Participants mean (SD) age 67.4 (6.0) years, disease duration 17 (NSD) years, BMI 26.8 (3.9) kgm^2^, *n* = 34 (M:F = 2:32) | ACR criteria for hand OA ^9^ |
| Kortekaas ^17^ | Investigate the association between features of US detected inflammation and development of erosive disease in patients with hand OA over 2.3 years of follow-up | Longitudinal cohort study design. Participants mean (SD) age 61.2 (8.9) years, disease duration 4 (NSD) years, BMI 27.6 (4.6), *n* = 56 (M:F = 8:48) | ACR criteria for OA ^9^ |
| Mathiessen ^18^ | To examine whether US predicts radiographic hand OA progression after 5 years | Longitudinal cohort study. Participants mean (SD) age 67.8 (5.2) years, disease duration 18.5 (7.9) years, BMI 25.4 (3.7), *n* = 78 (M:F = 7:71) | ACR criteria for OA ^9^ |
| Kortekaas ^19^ | Investigate whether inflammatory US features are associated with structural radiographic damage after long-term follow-up of 2-3 years and to investigate the course of inflammatory ultrasound features over long-term follow-up | Longitudinal observational cohort study design Participants mean (SD) age 61.2 (8.9) years, BMI 27.6 (4.6), *n* = 56 (M:F = 8:48) | ACR criteria for hand OA ^9^ |
| Mancarella ^20^ | To evaluate the association between US detected inflammation at baseline and subsequent development of new bone erosions at follow-up in patients with hand OA | Case control study design. Participants of the Oslo hand OA cohort  **Controls**  Participants mean (SD) age 66.8 (9.0) years, BMI 24.9 (3.4) kgm^2^, *n* = 10 (M:F = 2:8)  **Non-erosive OA**  Participants mean (SD) age 67.0 (7.5) years, BMI 25.8 (4.7) kgm^2^, *n* = 12 (M:F = 2:10)  **Erosive OA**  Participants mean (SD) age 63.9 (8.2) years, BMI 25.2 (2.9) kgm^2^, *n* = 13 (M:F = 0:13) | ACR criteria for hand OA ^9^ |

| **Author** | **Study aim** | **Study design/participant/sample size** | **Definition of OA cohort (inclusion criteria)** |
| --- | --- | --- | --- |
| Abraham  ^21^ | To measure the prevalence of features of OA in the dominant hand using US, within the Newcastle Thousand Families birth cohort | Prospective cohort study design. Participants mean (SD) age 63 (NSD) years, BMI 26.5 (4.2), *n* = 311 (M:F = 140:171) | The presence of at least one osteophyte in one hand joint |
| Kortekaas ^22^ | Investigate inflammatory US features and pain over a 3-month period in hand OA | Prospective cohort study design. Participants mean (SD) age 60 (8.8) years, BMI 28.0 (4.3),  *n* = 25 (M:F = 9:16) | ACR criteria for hand OA ^9^ |
| Usón ^23^ | To describe and compare the clinical, radiographic and US findings in patients with OA of the PIP and/or DIP joints with and without pain | Cross-sectional study design. Participants mean (SD) age 61.9 (NSD) years, disease duration 6.8 (NSD) years, *n* = 20 (M:F = 0:20) | ACR criteria for hand OA ^9^ |
| Kortekaas ^24^ | To compare inflammation as assessed by US between patients with the subset erosive hand OA versus non-erosive OA | Cross-sectional study design.  **Non-erosive OA**  Participants mean (SD) age 58.0 (8.9) years, BMI 26.9 (NSD) kgm2, *n* = 27 (M:F = 5:22)  **Erosive OA**  Participants mean (SD) age 65.0 (8.5) years, BMI 27.6 (NSD) kgm^2^, *n* = 28 (M:F = 3:25) | ACR criteria for hand OA ^9^ |
| Mathiessen ^25^ | To investigate the reliability of US assessment of osteophytes and clinical joint examination in patients with hand OA | Cohort study design. Participants mean (SD) age 68.6 (5.8) years, BMI 25.3 (3.6) kgm^2^ disease duration 18.3 (7.2) years, *n* = 127 (M:F = 11:116) | ACR criteria for hand OA ^9^ |
| Vlychou  ^26^ | To compare structural and inflammatory  features in small joints of the hand between patients with erosive OA and nodal hand OA by the use of high resolution USI and MRI | Case control study design.  **Non-erosive OA**  Participants mean (SD) age 62.0 (5.8) years, disease duration 5.9 (5.2) years, *n* = 7 (M:F = 0:7)  **Erosive OA**  Participants mean (SD) age 61.6 (8.6) years, disease duration 6.9 (4.4) years, *n* = 13 (M:F = 1:12)  **Controls**  Participants mean (SD) age 43.8 (4.3) years, *n* = 5 (M:F = 2:3) | ACR criteria for hand OA ^9^  Diagnosis of hand OA was further supported by a KL grade >2 in any IP joint  Nodal OA was defined as hand OA with Heberden’s or Bouchard’s nodes and no evidence of erosions on radiograph |

| **Author** | **Study aim** | **Study design/participant/sample size** | **Definition of OA cohort (inclusion criteria)** |
| --- | --- | --- | --- |
| Iagnocco ^27^ | To assess the reliability of US in detecting cartilage abnormalities at the MCPJ in people with cartilage pathology | Cross-sectional study design. Participants mean (SD) age 65 (NSD) years, disease duration 3.2 (NSD) years, *n* = 8 (M:F = 2:6) | ACR criteria for hand OA ^9^ |
| Arrestier ^28^ | To describe non-structural US abnormalities in finger OA to establish the prevalence of these abnormalities with healthy controls, and to evaluate correlations linking US abnormalities to clinical symptoms and radiographic damage | Case control study design.  **Cases**  Participants mean (SD) age 61.4 (8.6) years, disease duration 5 (NSD) years. *n* = 55 (M:F = 4:51)  **Controls**  Participants mean (SD) age 25.5 (4.3) years.  *n* = 46 (M:F = 13:33) | ACR criteria for finger OA ^9^ |
| Kortekaas ^29^ | Investigate the association between structural abnormalities on US and pain in hand OA | Cohort study design. Participants mean (SD) age 61.4 (9.3) years, BMI 27.7 (4.5) kgm^2^ disease duration 5.0 (NSD) years. *n* = 55 (M:F = 8:47) | ACR criteria for hand OA ^9^ |
| Kortekaas ^30^ | To investigate the association of US features: grey scale synovitis, synovial thickening, effusion, and PD signal with symptoms in hand OA | Cross-sectional study design. Participants mean (SD) age 62.0 (8.9) years, BMI 27.7 (4.5) kgm^2^ disease duration 5.0 (NSD) years. *n* = 55 (M:F = 7:48) | ACR criteria for hand OA ^9^ |
| Mancarella ^31^ | To examine US features of synovitis in hand OA joints, and to evaluate their relationship with radiological damage severity and US-detected cartilage thickness | Case control study deign.  **Controls**  Participants mean (SD) age 66.8 (9.0) years, BMI 24.9 (3.4) kgm^2^. *n* = 10 (M:F = 2:8)  **Non-erosive OA**  Participants mean (SD) age 67.0 (7.5) years, duration of disease 6 (NSD) years, BMI 25.8 (4.7) kgm^2^. *n* = 12 (M:F = 2:10)  **Erosive OA**  Participants mean (SD) age 63.9 (8.2) years, duration of disease 7 (NSD) years, BMI 25.2 (2.9) kgm^2^. *n* = 13 (M:F = 0:13) | ACR criteria for hand OA ^9^ |

| **Author** | **Study aim** | **Study design/participant/sample size** | **Definition of OA cohort (inclusion criteria)** |
| --- | --- | --- | --- |
| Vlychou ^32^ | Compare sonographic and radiographic imaging for the detection of erosions and osteophytes in hand joints of erosive OA patients and evaluate  additional sonographic findings using grey scale and PD imaging | Cross-sectional study design. Participants mean (SD) age 62.5 (NSD) years, duration of disease 4.2 (NSD) years. *n* = 22 (M:F = 2:20) | ACR criteria for hand OA ^9^ |
| Keen ^33^ | To develop a preliminary US hand OA scoring system, initially focusing on relevant pathological features with potentially high reliability | Cross-sectional study design. Seven participants no further information was reported. | NR |
| Keen ^34^ | Compare the detection of osteophytosis and joint space narrowing by US and radiographic in hand OA | Cross-sectional study design. Participants median (IQR) age 57 (53-66) years, duration of disease 4.2 (2.7-7.8) years. *n* = 37 (M:F = 6:31) | ACR criteria for hand OA ^9^ OR  with symptoms and radiographic structural changes consistent with OA such as sclerosis, joint space narrowing or osteophytes. |
| Keen ^35^ | Determine the extent of US detected pathology and investigate its relationship with symptoms in hand OA | Case control study deign.  **OA group**  Participants mean (SD) age 58 (NSD) years.  *n* = 36 (M:F = 5:31)  **Controls**  Participants mean (SD) age 58 (NSD) years.  *n* = 19 (M:F = 6:13) | ACR criteria ^9^ OR had radiographic changes consistent with hand OA who reported hand pain. |

US, Ultrasound; USI, Ultrasound imaging; OA, Osteoarthritis; PD, power Doppler; ACR, American College of Rheumatology; KL, Kellgren and Lawrence; M, Male; F, Female; BMI, Body mass index; SD, Standard deviation; NSD, No standard deviation; IQR, Interquartile range; NR, Not reported

1. Zabotti A, Filippou G, Canzoni M, Adinolfi A, Picerno V, Carrara G, et al. OMERACT agreement and reliability study of ultrasonographic elementary lesions in osteoarthritis of the foot. RMD open 2019; 5: e000795.

2. Menz HB, Munteanu SE, Landorf KB, Zammit GV, Cicuttini FM. Radiographic classification of osteoarthritis in commonly affected joints of the foot. Osteoarthritis and cartilage 2007; 15: 1333-1338.

3. Iagnocco A, Filippucci E, Riente L, Meenagh G, Delle Sedie A, Sakellariou G, et al. Ultrasound imaging for the rheumatologist XXXV. Sonographic assessment of the foot in patients with osteoarthritis. 2011.

4. Fjellstad CM, Mathiessen A, Slatkowsky-Christensen B, Kvien TK, Hammer HB, Haugen IK. Associations Between Ultrasound-Detected Synovitis, Pain, and Function in Interphalangeal and Thumb Base Osteoarthritis: Data From the Nor-Hand Cohort. Arthritis care & research 2020; 72: 1530-1535.

5. Steen Pettersen P, Neogi T, Magnusson K, Hammer HB, Uhlig T, Kvien TK, et al. Associations Between Radiographic and Ultrasound‐Detected Features in Hand Osteoarthritis and Local Pressure Pain Thresholds. Arthritis & rheumatology 2020; 72: 966-971.

6. Besselink NJ, Jacobs JWG, Westgeest AAA, van der Meijde P, Welsing PMJ, Marijnissen ACA, et al. Can optical spectral transmission assess ultrasound-detected synovitis in hand osteoarthritis? PLoS One 2019; 14: e0209761.

7. Oo WM, Deveza LA, Duong V, Fu K, Linklater JM, Riordan EA, et al. Musculoskeletal ultrasound in symptomatic thumb-base osteoarthritis: clinical, functional, radiological and muscle strength associations. BMC musculoskeletal disorders 2019; 20: 220.

8. Kroon FPB, van Beest S, Ermurat S, Kortekaas MC, Bloem JL, Reijnierse M, et al. In thumb base osteoarthritis structural damage is more strongly associated with pain than synovitis. Osteoarthritis and cartilage 2018; 26: 1196-1202.

9. Altman R, Alarcon G, Appelrouth D, Bloch D, Borenstein D, Brandt K, et al. The American College of Rheumatology criteria for the classification and reporting of osteoarthritis of the hand. Arthritis & Rheumatism: Official Journal of the American College of Rheumatology 1990; 33: 1601-1610.

10. Sivakumaran P, Hussain S, Ciurtin C. Comparison between Several Ultrasound Hand Joint Scores and Conventional Radiography in Diagnosing Hand Osteoarthritis. Ultrasound in medicine & biology 2018; 44: 544-550.

11. Zhang W, Doherty M, Leeb B, Alekseeva L, Arden N, Bijlsma J, et al. EULAR evidence-based recommendations for the diagnosis of hand osteoarthritis: report of a task force of ESCISIT. Annals of the rheumatic diseases 2009; 68: 8-17.

12. Magnusson K, Mathiessen A, Hammer HB, Kvien TK, Slatkowsky-Christensen B, Natvig B, et al. Smoking and alcohol use are associated with structural and inflammatory hand osteoarthritis features. Scandinavian journal of rheumatology 2017; 46: 388-395.

13. Mathiessen A, Slatkowsky-Christensen B, Kvien TK, Haugen IK, Hammer HB. Ultrasound-detected osteophytes predict the development of radiographic and clinical features of hand osteoarthritis in the same finger joints 5 years later. RMD Open 2017; 3: e000505-e000505.

14. Spolidoro Paschoal NdO, Natour J, Machado FS, Alcântara Veiga de Oliveira H, Vilar Furtado RN. Interphalangeal Joint Sonography of Symptomatic Hand Osteoarthritis: Clinical and Functional Correlation. Journal of ultrasound in medicine : official journal of the American Institute of Ultrasound in Medicine 2017; 36: 311-319.

15. Hammer HB, Iagnocco A, Mathiessen A, Filippucci E, Gandjbakhch F, Kortekaas MC, et al. Global ultrasound assessment of structural lesions in osteoarthritis: a reliability study by the OMERACT ultrasonography group on scoring cartilage and osteophytes in finger joints. Annals Of The Rheumatic Diseases 2016; 75: 402-407.

16. Haugen IK, Mathiessen A, Slatkowsky-Christensen B, Magnusson K, Bøyesen P, Sesseng S, et al. Synovitis and radiographic progression in non-erosive and erosive hand osteoarthritis: is erosive hand osteoarthritis a separate inflammatory phenotype? Osteoarthritis and cartilage 2016; 24: 647-654.

17. Kortekaas MC, Kwok WY, Reijnierse M, Stijnen T, Kloppenburg M. Brief Report: Association of Inflammation With Development of Erosions in Patients With Hand Osteoarthritis: A Prospective Ultrasonography Study. Arthritis & Rheumatology 2016; 68: 392-397.

18. Mathiessen A, Slatkowsky-Christensen B, Kvien TK, Hammer HB, Haugen IK. Ultrasound-detected inflammation predicts radiographic progression in hand osteoarthritis after 5 years. Annals of the rheumatic diseases 2016; 75: 825-830.

19. Kortekaas MC, Kwok W-Y, Reijnierse M, Kloppenburg M. Inflammatory ultrasound features show independent associations with progression of structural damage after over 2 years of follow-up in patients with hand osteoarthritis. Annals Of The Rheumatic Diseases 2015; 74: 1720-1724.

20. Mancarella L, Addimanda O, Pelotti P, Pignotti E, Pulsatelli L, Meliconi R. Ultrasound detected inflammation is associated with the development of new bone erosions in hand osteoarthritis: a longitudinal study over 3.9 years. Osteoarthritis and cartilage 2015; 23: 1925-1932.

21. Abraham AM, Pearce MS, Mann KD, Francis RM, Birrell F. Population prevalence of ultrasound features of osteoarthritis in the hand, knee and hip at age 63 years: the Newcastle thousand families birth cohort. BMC musculoskeletal disorders 2014; 15: 162.

22. Kortekaas MC, Kwok WY, Reijnierse M, Huizinga TW, Kloppenburg M. Follow-up study of inflammatory ultrasound features in hand osteoarthritis over a period of 3 months: variable as well as constant. Osteoarthritis & Cartilage 2014; 22: 40-43.

23. Usón J, Fernández-Espartero C, Villaverde V, Condés E, Godo J, Martínez-Blasco MJ, et al. Symptomatic and asymptomatic interphalageal osteoarthritis: An ultrasonographic study. Reumatologia clinica 2014; 10: 278-282.

24. Kortekaas MC, Kwok WY, Reijnierse M, Huizinga TW, Kloppenburg M. In erosive hand osteoarthritis more inflammatory signs on ultrasound are found than in the rest of hand osteoarthritis. Annals of the Rheumatic Diseases 2013; 72: 930-934.

25. Mathiessen A, Haugen IK, Slatkowsky-Christensen B, Bøyesen P, Kvien TK, Hammer HB. Ultrasonographic assessment of osteophytes in 127 patients with hand osteoarthritis: exploring reliability and associations with MRI, radiographs and clinical joint findings. Annals of the rheumatic diseases 2013; 72: 51-56.

26. Vlychou M, Koutroumpas A, Alexiou I, Fezoulidis I, Sakkas LI. High-resolution ultrasonography and 3.0 T magnetic resonance imaging in erosive and nodal hand osteoarthritis: high frequency of erosions in nodal osteoarthritis. Clinical rheumatology 2013; 32: 755-762.

27. Iagnocco A, Conaghan P, Aegerter P, Möller I, Bruyn G, Chary-Valckenaere I, et al. The reliability of musculoskeletal ultrasound in the detection of cartilage abnormalities at the metacarpo-phalangeal joints. Osteoarthritis and cartilage 2012; 20: 1142-1146.

28. Arrestier S, Rosenberg C, Etchepare F, Rozenberg S, Foltz V, Fautrel B, et al. Ultrasound features of nonstructural lesions of the proximal and distal interphalangeal joints of the hands in patients with finger osteoarthritis. Joint, bone, spine: revue du rhumatisme 2011; 78: 65-69.

29. Kortekaas MC, Kwok WY, Reijnierse M, Huizinga TW, Kloppenburg M, Kortekaas MC, et al. Osteophytes and joint space narrowing are independently associated with pain in finger joints in hand osteoarthritis. Annals of the Rheumatic Diseases 2011; 70: 1835-1837.

30. Kortekaas MC, Kwok WY, Reijnierse M, Watt I, Huizinga TW, Kloppenburg M, et al. Pain in hand osteoarthritis is associated with inflammation: the value of ultrasound. Annals of the rheumatic diseases 2010; 69: 1367-1369.

31. Mancarella L, Magnani M, Addimanda O, Pignotti E, Galletti S, Meliconi R. Ultrasound-detected synovitis with power Doppler signal is associated with severe radiographic damage and reduced cartilage thickness in hand osteoarthritis. Osteoarthritis And Cartilage 2010; 18: 1263-1268.

32. Vlychou M, Koutroumpas A, Malizos K, Sakkas LI, Vlychou M, Koutroumpas A, et al. Ultrasonographic evidence of inflammation is frequent in hands of patients with erosive osteoarthritis. Osteoarthritis and cartilage 2009; 17: 1283-1287.

33. Keen HI, Lavie F, Wakefield RJ, D'Agostino MA, Hammer HB, Hensor E, et al. The development of a preliminary ultrasonographic scoring system for features of hand osteoarthritis. Annals Of The Rheumatic Diseases 2008; 67: 651-655.

34. Keen HI, Wakefield RJ, Grainger AJ, Hensor EM, Emery P, Conaghan PG. Can ultrasonography improve on radiographic assessment in osteoarthritis of the hands? A comparison between radiographic and ultrasonographic detected pathology. Annals of the rheumatic diseases 2008; 67: 1116-1120.

35. Keen HI, Wakefield RJ, Grainger AJ, Hensor EM, Emery P, Conaghan PG. An ultrasonographic study of osteoarthritis of the hand: synovitis and its relationship to structural pathology and symptoms. Arthritis care & research 2008; 59: 1756-1763.
