# Supplementary material for: Evaluation of osteoarthritic features in peripheral joints by ultrasound imaging: A systematic review
Source: Osteoarthr Cartil Open. 2021 Jul 16;3(3):100194. doi: 10.1016/j.ocarto.2021.100194 (PMC9718269; doi:10.1016/j.ocarto.2021.100194)
Supplement: Multimedia component 2 [file mmc2.docx]

| **Evaluation of osteoarthritic features in peripheral joints by ultrasound imaging: a systematic review**  **Supplementary Material 2: Defining and grading sonographic features of osteoarthritis** | | | | | | | | | |
| --- | --- | --- | --- | --- | --- | --- | --- | --- | --- |
| **Author** | **Joint** | **USI feature** | **Definition** | **Atlas included** | **Origin of USI atlas** | **Grading system** | **USI acquisition protocol** | **Sonographer** | **Reliability** |
| Zabotti ^1^ | Foot:  First MTPJ  Midfoot | Synovial hypertrophy | NR | No | NA | Absent /present ^2^ | NR | 11 rheumatologists all experts in US and members of OMERACT group | Intraobserver reliability  ĸ = 0.64  Interobserver round 1  ĸ = 0.50  Interobserver round 2  ĸ = 0.64 |
|  |  |  |  |  |  | Semiquantitative (0-3)  ^3^ |  |  | Intraobserver reliability  ĸ = 0.48  Interobserver round 1  ĸ = 0.63  Interobserver round 2  ĸ = 0.59 |
|  |  | PD Signal | NR |  |  | Semiquantitative (0-3)  ^3^ |  |  | Intraobserver reliability  ĸ = 0.90  Interobserver round 1  ĸ = 0.87  Interobserver round 2  ĸ = 0.89 |
|  |  | Joint effusion | NR |  |  | Absent /present ^2^ |  |  | Intraobserver reliability  ĸ = 0.67  Interobserver round 1  ĸ = 0.80  Interobserver round 2  ĸ = 0.61 |
|  |  | Osteophytes | NR |  |  | Semiquantitative (0-3)  0 = none  1 = minor  2 = moderate  3 = major size of osteophytes  ^4^ |  |  | Intraobserver reliability  ĸ = 0.63  Interobserver round 1  ĸ = 0.54  Interobserver round 2  ĸ = 0.58 |
|  |  | Cartilage damage  First MTPJ only due to probe positioning | Loss of anechoic structure and/or thinning of cartilage layer ^5^ |  |  | Absent/ present ^5^ |  |  | Intraobserver reliability  ĸ = 0.64  Interobserver round 1  ĸ = 0.60  Interobserver round 2  ĸ = 0.63 |
| Iagnocco Iagnocco, et al. ^6^ | Foot:  STJ  TNJ  NCJ-M  NCJ-I  MTPJ 1-5 | Joint effusion | Abnormal hypoechoic or anechoic intra-articular material that is displaceable and compressible but does not exhibit PD signal ^7^ | No | NA | All lesions were registered according to a dichotomous (present of absent) score | Loqiq9 machine equipped with a multi-frequency linear probe, operating at 14 MHz.  According to the EULAR guidelines for MSK  US in rheumatology, in all cases, longitudinal and transverse multiplanar scans were performed at the level of the dorsal, lateral and medial aspects of the foot ^8^ | Single sonographer  who was a rheumatologist experienced in MSK US and was blinded to the clinical and laboratory findings. | NR |
|  |  | Synovial hypertrophy | Abnormal hyopechoic intraarticular tissue that is nondisplaceable and poorly compressible and which may exhibit PD signal ^7^ |  |  |  |  |  |  |
|  |  | PD vascularisation | NR |  |  |  |  |  |  |
|  |  | Osteophytes | Cortical protrusion at the joint margin seen in two planes ^9^ |  |  |  |  |  |  |

| **Hand OA** | | | | | | | | | |
| --- | --- | --- | --- | --- | --- | --- | --- | --- | --- |
| **Author** | **Joint** | **USI feature** | **Definition** | **Atlas included** | **Origin of USI atlas** | **Grading system** | **USI acquisition protocol** | **Ultrasonographer** | **Reliability** |
| Fjellstad ^10^ | Hand:  DIP  PIP  CMC 1 | Synovitis | NR | Yes | NR | Semiquantitative (0–3) ^11^ | The joints were scanned dorsally with longitudinal projection, from the radial to the ulnar side. An  additional transverse scanning was carried out when presence of  pathology was uncertain | A trained medical student performed the US examination. Scoring was performed in consensus with an experienced ultrasonographer | DIP/PIP ĸ = 0.80  CMC1 ĸ = 0.92 |
|  |  | PD Signal | NR |  |  |  |  |  | DIP/PIP ĸ = 0.85  CMC1 ĸ = 0.92 |
|  |  | Osteophytes | NR |  |  |  |  |  | DIP/PIP ĸ = 0.72  CMC1 ĸ = 0.80 |
| Steen Pettersen ^12^ | Hand:  DIP  PIP | Synovitis | NR | No | NA | Semiquantitative (0-3) ^11^ | All hand joints were scanned dorsally with longitudinal projection from the radial to the ulnar side of each joint. An additional transverse scan was performed when the presence of pathologic features of OA was uncertain | A trained medical  student performed the US examinations. Initial scorings were done in consensus with an experienced ultrasonographer | Inter-reader reliability of  the assessments of the DIP/PIP joints in 10 participants between  the medical student and the ultrasonographer was good  (κ = 0.80 for gray-scale synovitis grades 0–3) |
|  |  | PD activity | NR |  |  | Semiquantitative (0-3)  Due to the low frequency of grade (2–3) PD activity, this variable was later dichotomised  Grade 0 versus grades (1-3) ^11^ |  |  | Inter-reader reliability was good for the absence/presence of PD activity (κ = 0.79) |
| Besselink ^13^ | Hand:  DIP 2-5  PIP 1-5  CMC 1  MCP 1 | US Synovitis | Refers to previous manuscript ^14^ | No | NA | Semiquantitative (0-3) ^2^ | Mylab 60 system (Esaote, GEN  Patient and probe  positioning were performed according to EULAR guidelines ^15^ | US examination was performed by a single experienced examiner | NR |
|  |  | PD synovitis | Refers to previous manuscript ^14^ |  |  | Semiquantitative (0-3) ^2^ |  |  | NR |
|  |  | Osteophytes | Refers to previous manuscript ^14^ |  |  | Absent /present |  |  | NR |
|  |  | Erosions | Refers to previous manuscript ^14^ |  |  | Absent /present |  |  | NR |
| Oo ^16^ | Hand: CMC | Synovitis (effusion and synovial hypertrophy) | Effusion was defined as hypoechoic or anechoic fully compressible material  Synovial hypertrophy was defined as echogenic or hypoechoic slightly compressible or  non-compressible intra-articular tissue ^17^ | No | NA | Synovial hypertrophy and effusion were considered together as a single domain “synovitis”  0 = absent  1 = mild  2 = moderate  3 = severe ^18^ | The thumb-base joint was scanned on the longitudinal and transverse plane of the palmar and dorsal aspect according to the OMERACT ultrasound definitions and scanning methods ^11^ | Sonographer, experience in MSK US | Intra-rater reliability  ĸw = 0.77 |
|  |  | PD signal | Doppler signal as a pulsating colour spot found within the synovial structure ^7^ | No | NA | Absent /present ^7^ |  |  | Intra-rater reliability  unweighted  ĸ = 0.89 |
|  |  | Osteophytes | cortical protrusions at the joint margin seen in two planes ^7^ | Yes | ^4^ | Semi-quantitatively using an atlas (0–3), based on the largest osteophyte ^4^ |  |  | Intra-rater reliability  ĸ = 0.79 |
|  |  | Erosions | Intra-articular discontinuity of the bone surface that is visible in two perpendicular planes ^7^ | No | NA | Absent /present |  |  | NR |
| Kroon ^19^ | Hand: CMC | Synovial thickening | NR | No | NA | semiquantitative scale:  0 = none  1 = mild  2 = moderate  3 = severe ^20^ | NR | Two ultrasonographers, who scored together in consensus during the examination | NR |
|  |  | Joint effusion | NR |  |  |  |  |  | NR |
|  |  | PD signal | NR |  |  |  |  |  | NR |
| Sivakumaran ^21^ | Hand:  1^st^ CMC  MCP  PIP  DIP | PD signal | Active joint inflammation refers to ^22^ | No | NR | Semiquantitative (1-3) ^23^ | Dorsal longitudinal  and transverse views of wrists and MCP, PIP, DIP  and carpometacarpal 1 [CMC-1] joints | Clinician with 6 years’ experience in US. | NR |
|  |  | Synovitis | NR but refers to ^23^ |  |  | Semiquantitative (1-3) ^23^ |  |  |  |
|  |  | Synovial thickening | NR but refers to ^23^ |  |  | Semiquantitative (1-3) ^23^ |  |  |  |
|  |  | Joint effusion | NR but refers to ^23^ |  |  | Present or absent ^23^ |  |  |  |
|  |  | Erosions | An intra-articular discontinuity of the bone  surface that is visible in two perpendicular planes ^7^ |  |  | Present or absent ^7^ |  |  |  |
|  |  | Osteophytes | Hyperechoic signal in the area of the attachment  of the joint capsule to the bony cartilaginous margin that correspond with the eventual appearance of  osteophytes visualised on the PR ^24^ |  |  | Present or absent |  |  |  |
| Magnusson ^25^ | Hand:  CMC 1  MCP 1-5  PIP 2-5  DIP 2-5 | Synovitis | NR | Yes | USI RA atlas was used as a reference ^17^ | Present or absent  A previously published atlas for RA was used as a reference ^17^ | NR | Three medical students trained in USI | NA |
| Mathiessen ^26^ | Hand:  CMC 1  IP 1  PIP 2-5  DIP 2-5 | Osteophytes | NR | No | NA | Semiquantitative (0–3) ^4^ | Longitudinal and transverse US examination was performed on both hands on the volar and dorsal sides | Two sonographers performed assessments and reached consensus on each scoring  They were the same sonographers from Mathiessen, et al. ^4^ and Mathiessen, et al. ^27^ | NR |
| Spolidoro Paschoal ^28^ | Hand:  DIP  PIP | Synovial hypertrophy | NR | No | NA | Semiquantitative  0 = no fluid  1 = a minimal amount  of fluid  2 = a moderate amount of fluid (without distension of the joint capsule)  3 = extensive amount of fluid (with distension of the joint capsule) ^29^ | NR | Two rheumatologists experienced in MSK US | Distal joint:  Intraobserver reliability  Dorsal: ĸ = 0.673  Palmer: ĸ = -0.044  Interobserver reliability  Dorsal: ĸ = 0.617  Palmer: ĸ = 0.498  Proximal joint: Intraobserver reliability  Dorsal: ĸ = 0.664  Palmer: ĸ = 0.640  Interobserver reliability  Dorsal: ĸ = 0.390  Palmer: ĸ = 0.525 |
|  |  | Synovial blood flow (PD signal) | NR |  |  | Semiquantitative  0 = no flow in the synovia  1 = single vessel signals  2 = confluent-vessel signals in less than half of the area of the synovia  3 = vessel signals in more than half of the area of the synovia ^7, 29^ |  |  | Power Doppler signals were found in only 1.7% of the sample, precluding statistical analysis. |
| Hammer ^30^ | Hand:  CMC 1  MCP 1-5  PIP 1-5  DIP 2-5 | Osteophyte | Formation of excess bone at the joint margins | Yes | USI atlas was used as a reference ^4^ | Semiquantitative  0 = none  1 = minor  2 = moderate  3 = major size of osteophytes ^4^ | Five identical General Electric logic E9 machines (GE Medical Systems, Milkaukee, Wisconsin, USA), equipped with two multifrequency linear probes (hockey stick 8-18MHz used for scoring cartilage and regular probe 6-15 MHz used for scoring osteophytes)  Osteophytes were assessed by longitudinal scanning on extended joints with swiping of the probe from side to side of the dorsal aspect of MCP, PIP and DIP joints and at the radiopalmar  side of CMC1 joints.  For the evaluation of cartilage, the MCP joints were kept in maximal flexion (ie, close to 90°) and a longitudinal dorsal scan was performed at the level of the mid portion of the metacarpal head | 10 sonographers (9 were rheumatologists, experts in MSK USI and members of the OMERACT US group and one trainee fellow in rheumatology, highly experienced and had participated in the development of the USI atlas | Osteophyte scores were evenly distributed, and the intraobserver and interobserver reliabilities were substantial to excellent  κ range = (0.68–0.89) mean  Day 1 κ = 0.65  Day 2 κ = 0.67 |
|  |  | Cartilage | NR |  | Developed new USI atlas for scoring cartilage damage | Semiquantitative  0 = normal cartilage  1 = loss of anechoic structure and/or focal thinning of  cartilage layer OR irregularities and/or loss of sharpness of at least one cartilage margin;  2 = loss of anechoic structure and/or focal thinning of  cartilage layer AND irregularities and/or loss of sharpness of at least one cartilage margin;  3 = focal absence or complete loss of the cartilage |  |  | Cartilage scores were unevenly distributed, and the intraobserver and interobserver reliability was fair to moderate  κ range = (0.46–0.66) mean:  Day 1 κ = 0.39  Day 2 κ = 0.33 |
| Haugen ^31^ | Hand:  DIP 2-5  PIP 1-5 | Synovitis | grey-scale synovitis,  including both thickened synovium and fluid | Yes | USI RA atlas was used as a reference ^17^ | Semiquantitative  0 = none  1 = minor  2 = moderate  3 = major presence of US pathology ^17^ | Each joint was scanned longitudinally from the radial to the ulnar side, and transverse scanning was performed if there was uncertainty about the presence of pathology ^4^ | A trained medical student and an experienced rheumatologist performed the US assessments  together and reached consensus on each scoring | NR |
|  |  | PD activity | Presence of vascularisation |  |  | Semiquantitative  0 = none  1 = minor  2 = moderate  3 = major presence of US pathology ^17^ |  |  | NR |
| Kortekaas ^32^ | Hand:  All DIPJ  All PIPJ | PD signal | NR | No | NA | Semiquantitative  0 = none  1 = mild  2 = moderate  3 = severe ^20^ | NR | One experienced ultrasonographer while always in the presence of a second ultrasonographer,  scoring together in consensus | NR |
|  |  | Synovial thickening | NR |  |  |  |  |  | NR |
|  |  | Joint Effusion | NR |  |  |  |  |  | NR |
| Mathiessen ^27^ | Hand:  CMC 1  MCP 1-5  PIP 1-5  DIP 2-5 | Synovitis | A combined score of thickened synovium and joint effusion | Yes | ^17^ | Semiquantitative  0 = none  1 = minor  2 = moderate  3 = major presence of US  Pathology ^17^ | Each joint was scanned longitudinally from the radial to the ulnar side, and transverse scanning was performed if there was uncertainty about the presence of pathology | One trainee and one experienced rheumatologist  performed the US assessments together and reached consensus on each scoring | Good inter-reader reliability for synovitis  (κw = 0.74)  Intra-reader reliability was very good for both features  (κw > 0.86) |
|  |  | PD activity | Represented presence of vascularisation |  |  |  |  |  | Inter-reader reliability  for PD (κw > 0.93) |
| Kortekaas ^33^ | Hand:  All DIPJ  All PIPJ  1^st^ IPJ  1^st^ CMC  MCP | Synovial thickening | NR | No, but radiographic progression of  osteophytes and joint space narrowing were scored using the OARSI atlas | ^34^ | Semiquantitative  0 = none  1 = mild  2 = moderate  3 = severe ^20^ | Hand joints were scanned on the dorsal side in longitudinal and  transverse planes | One experienced ultrasonographer, scoring together in consensus with a second ultrasonographer. Both blinded to clinical findings | Intraobserver reliability was tested by performing a second ultrasound in 10% (randomly chosen) of patients  ICC = 0.93 |
|  |  | Effusion |  |  |  |  |  |  | ICC = 0.84 |
|  |  | PD activity |  |  |  |  |  |  | ICC = 0.62 |
| Mancarella ^35^ | Hand:  PIP 1-5  DIP 2-5 | PD activity | A signal within a region of GS synovitis | No | NA | Present or absent ^7^ | Longitudinal and transverse US examination was performed on both hands on the volar and dorsal sides | Two experienced MSK sonographers | Intra-observer variability depicted by ĸ coefficient was 0.78 |
|  |  | Synovial thickening | GS synovitis |  |  | Present or absent ^7^ |  |  | Intra-observer variability depicted by ĸ coefficient was 0.84 |
|  |  | Joint effusion | OMERACT definitions ^7^ |  |  | Present or absent ^7^ |  |  | Intra-observer variability depicted by ĸ coefficient was 0.83 |
|  |  | Erosions | An intra-articular discontinuity of the bone  surface that is visible in two perpendicular planes on imaging ^7^ |  |  | NR |  |  | Intra-observer variability depicted by ĸ coefficient was 0.87 |
| Abraham ^36^ | Hand:  1^st^ CMC  Index:  MCP  PIP  DIP | Osteophytes | Cortical protrusions seen in two planes ^37^ | No | NA | Present or absent  (Present if at least one osteophyte in the individual joint) | Mylab 70 XVG machine (ESAOTE, Genoa, Italy).  Dominant hand imaged using a 10-18 MHz linear transducer  Dynamic approach with the probe in a longitudinal position and being swept across the whole of the joint for DIP and PIP joints from the anterior to posterior aspect, and across accessible areas for the MCP and CMC joints. The hand joints were placed in a neutral position for all examinations | Trained MSK Ultrasonographers | The ĸ inter-rater reliability for HOA was moderate to substantial, with values ranging from 0.50 to 0.69 |
| Kortekaas ^38^ | Hand:  CMC 1  MCP 1-5  PIP 1-5  DIP 2-5 | PD signal | As described previously ^20^ | No | NA | All ultrasound features were scored on a 4-point semiquantitative scale:  0 = none  1 = mild  2 = moderate  3 = severe ^20^ | Scanned on the dorsal side in longitudinal and transverse planes | US assessment by one ultrasonographer and scored together with a second ultrasonographer | NR |
|  |  | Synovial thickening |  |  |  |  |  |  | NR |
|  |  | effusion |  |  |  |  |  |  | NR |
| Usón ^39^ | Hand:  PIP  DIP | Osteophytes | Hyperechoic cortical protrusions visualised in two planes | No | NA | Absent/present | A General Electric Logic 9 ultrasoundmachine with an M12 linear probe.  In their longitudinal and transverse axis, the dorsal, palmar, lateral and medial aspects of each PIP and DIP joint, with the hand outstretched on the table. | An ultrasound expert rheumatologist | NR |
|  |  | Joint impingement | Decrease in the space between the cortical margins |  |  | Absent/present |  |  | NR |
|  |  | US synovitis (effusion and/or synovial hypertrophy) | Distension of the joint capsule ≥1.5 mm in its anteroposterior diameter with compressible material |  |  | Absent/present |  |  | NR |
|  |  | PD signal | Intraarticular Doppler signal |  |  | Absent/present |  |  | NR |
|  |  | Erosions | Intraarticular cortical defect visualised in three planes |  |  | Absent/present |  |  | NR |
|  |  | Cartilage | The display or non-display of an anechoic band over the head of the phalanx was assessed |  |  | Absent/present |  |  | NR |
| Kortekaas ^40^ | Hand:  PIP  DIP | PD signal | NR | No | NA | All ultrasound features were scored on a 4-point Semiquantitative scale:  0 = none  1 = mild  2 = moderate  3 = severe  Synovial thickening and  effusion were scored in accordance with the scoring system for  inflammatory signs in RA ^29^ | Scanned from the dorsal and lateral side only in longitudinal and transverse planes, in accordance with a group of experts in order to develop a scoring system for ultrasound for hand OA ^11^ Features had to be present in both planes | US assessment by one ultrasonographer and scored together with a second ultrasonographer | Intra-observer variability was tested by performing a second ultrasound in 10% (five) of all patients.  Intra-observer variability  ICC = 0.57 |
|  |  | Effusion | The definition of synovial thickening and effusion followed the outcome measures in rheumatoid arthritis clinical trials definitions ^7^ |  |  |  |  |  | Intra-observer variability  ICC = 0.73 |
|  |  | Synovial thickening |  |  |  |  |  |  | Intra-observer variability  ICC = 0.73 |
|  |  | Osteophytes | NR |  |  |  |  |  | Intra-observer variability  ICC = 0.71 |
| Mathiessen ^4^ | Hand:  CMC 1  MCP 1-5  PIP 1-5  DIP 2-5 | Osteophyte | Cortical protrusions ^7^ | Developed new USI atlas of osteophytes | Trainee sonographer collected still images from US exam and developed novel US atlas | Semiquantitative  0 = none  1 = minor  2 = moderate  3 = major size of osteophytes ^17, 18^ | A linear array transducer was used 5–13 MHz,  Each joint was scanned longitudinally from the radial to the ulnar side, and transverse scanning  was performed if there was uncertainty about the presence of pathology | One trainee and one experienced sonographer  performed the ultrasound assessments together and reached consensus on each scoring | Excellent intra and inter-reader reliability for both readers and scoring sessions (κw > 0.91) |
| Vlychou ^41^ | Hand:  MCP  PIP  DIP | Effusion | Completely transonic, compressible, and with no increase in PD signal | No | NA | Present/absent | Protocol included transverse and longitudinal scanning ^42^  The sonographic scanning process and the definitions of findings have been published previously ^43^ | Radiologist experienced in MSK US | Agreement between US and MRI for features of hand OA  ĸ = 0.87 |
|  |  | Osteophytes | Intra-articular discontinuity of the bone surface that is visible in two perpendicular planes; bone proliferation  is osseous proliferation of the cortex in the area adjacent to the joint |  |  |  |  |  | Agreement between US and MRI for features of hand OA  ĸ = 0.79 |
|  |  | Synovitis | An anechoic or hypoechoic intra-capsular area, different from cartilage with or without PD signal |  |  |  |  |  | Agreement between US and MRI for features of hand OA  ĸ = 0.82 |
|  |  | Tenosynovitis | A hypoechoic rim around tendon with or without PD signal |  |  |  |  |  | Agreement between US and MRI for features of hand OA  ĸ = 0.83 |
|  |  | Erosions | NR |  |  |  |  |  | Agreement between US and MRI for features of hand OA  ĸ = 0.84 |
| Iagnocco ^5^ | 2-5 MCP | Cartilage | Loss of anechoic structure and/or thinning of cartilage layer, and irregularities and/or loss of sharpness of at least one cartilage margin | No, but consensus was obtained on image interpretation of normal and pathological US findings from static images during a training session | Delphi method to reach consensus on which definitions they would recommend for testing the reliability of US in hand OA | Present/ absent  In addition, the following basic lesions were evaluated: loss of anechoic structure and/or thinning of the cartilage layer, and irregularities and/or loss of sharpness of at least one cartilage margin | Eight identical MyLab 70 X-Vision gold machines, equipped with a mulit-frequency (6-8 MHz) linear probe operating at a frequency of 18 MHz ere used  Joints were examined  with a longitudinal dorsal scan, performed at the level of the median portion of the MCP joints | Nine rheumatologists, all experts in MSK USI. All members of the OMERACT US group and the OMERACT/OARSI US task force | Intra-observer κ values ranged from 0.52 - 1 for global cartilage abnormalities  κ values ranged from 0.54 - 0.94 for loss of anechoic structure and/or thinning of cartilage layer    κ values ranged from 0.59 - 1 for irregularities and/or loss of sharpness of at least one cartilage  margin    Values of ĸ for inter-observer reliability were 0.80 for global cartilage abnormalities, 0.62 for loss of anechoic structure and/or thinning of cartilage layer, and 0.39 for irregularities and/or loss of sharpness of at least one cartilage margin |
| Arrestier ^44^ | Hand:  2-5 PIP  2-5 DIP | Joint effusion | Echo-free zone,  OMERACT criteria ^7^ | No | NA | Semiquantitative  0 = no effusion  1 = effusion under the tendon  2 = moderate effusion without distension of the joint capsule  3 = large effusion with distension of the capsule ^29^ | Estaote (Technos MP) machine and a 10-13 MHz linear array transducer. Power Doppler was performed using a frequency of 8.3 MHz and a pulse repetition frequency of 750 MHz  Ultrasonography method recommended  by EULAR. The volar and palmar aspects of the  joints were scanned longitudinally | Two sonographers, both rheumatologists with over two years’ experience in OA USI | NR |
|  |  | PD signal | Hypervascularisation |  |  | semi-quantitatively  (0 - 3) |  |  |  |
|  |  | Synovitis | Intracapsular hypoechogenic zone, OMERACT criteria ^7^ |  |  | NR |  |  |  |
| Kortekaa^45^ | Hand:  CMC 1  MCP 1  IPJ 1-5  PIP 1-5  DIP 2-5 | Osteophytes | NR | No | NA | Semiquantitative  0 = none  1= mild  2 = moderate  3 = severe ^20^ | Scanned from the dorsal side only in the longitudinal and transverse planes, covering the dorsal and lateral sides of the joint, in accordance with a preliminary US scoring  system for hand OA ^11^ | Two ultrasonographers blinded to clinical findings and PR scores | Intraobserver variability was tested by performing a second US in 10% of randomly selected patients  Intraobserver variability  ICC= 0.71 |
|  |  | PD signal | NR |  |  |  |  |  | Intraobserver variability  ICC= 0.57 |
|  |  | Joint effusion | NR |  |  |  |  |  | Intraobserver variability  ICC= 0.73 |
|  |  | Synovial thickening | NR |  |  |  |  |  | Intraobserver variability  ICC= 0.73 |
| Kortekaa^20^ | Hand:  CMC 1  MCP 1  IPJ 1-5  PIP 1-5  DIP 2-5 | Synovitis | A composite of effusion and synovial thickening ^11^ | No | NA | Semiquantitative  0 = none  1 = mild  2 = moderate  3 = severe^11^ | Hand joints were scanned on the dorsal side in longitudinal and transverse planes ^11^ Features had to be present in both planes | Two ultrasonographers | Intraobserver variability was tested by performing a second US scan in 10% of randomly selected patients  NR for synovitis |
|  |  | Synovial thickening | Abnormal hypoechoic intra-articular material that is non-displaceable and poorly compressible and may exhibit PDS |  |  |  |  |  | Intraobserver variability  ĸ = 0.73 |
|  |  | Effusion | Abnormal hypoechoic or anechoic intra-articular material that is displaceable and compressible and does not exhibit PDS |  |  |  |  |  | Intraobserver variability  ĸ = 0.73 |
|  |  | PD signal | NR |  |  |  |  |  | Intraobserver variability  ĸ = 0.57 |
| Mancarella ^46^ | Hand:  MCP 1-5  PIP 1-5  DIP 2-5 | Synovitis (synovial hypertrophy and joint effusion) | Characterised by evaluating Synovial hypertrophy and effusion, using the OMERACT definitions developed for RA ^7^ | No | NA | Present/ absent ^18^ | Longitudinal and transverse US examination was performed on both hands on the volar and dorsal sides | Single sonographer experienced in MSK US, blinded to PR data | The intra-observer reliability was excellent with ĸ values of 0.910 for synovial hypertrophy |
|  |  |  |  |  |  |  |  |  | The intra-observer reliability was excellent with ĸ values of 0.943 for joint effusion |
|  |  | PD signal | A signal within a region of GS synovitis |  |  | Present/absent ^18^ |  |  | The intra-observer reliability was almost excellent with a ĸ value of 0.86 |
|  |  | Cartilage thickness | Well-defined anechogenic or homogeneously hypoechogenic band between the chondrosynovial and osteochondral margins ^24^ |  |  | Measured in mm |  |  | ICC for cartilage  thickness was excellent with a value of 0.926 |
| Vlychou ^43^ | Hand:  CMC  MCP  PIP  DIP | Osteophytes | Intra-articular discontinuity of the bone surface that is visible in two perpendicular planes; bone proliferation  is osseous proliferation of the cortex in the area adjacent to the joint | No | NA | Present/absent | Scanned using a multiplanar technique. Sagittal scans were performed in both volar and dorsal aspect of hand joints, complemented by axial views. PD US was applied in all joints, in order to detect the presence of inflamed synovium ^42^ | Trained radiologist with a 4-year experience in MSK US. Blinded to radiographic and clinical data | The intra-observer ĸ value for agreement for the sonographic detection of erosions and other findings was 0.81 |
|  |  | Erosions | According to Outcome  Measurement in Rheumatoid Arthritis (RA) and Connective Tissue  (OMERACT) |  |  |  |  |  |  |
|  |  | Joint effusion | A completely anechoic fluid collection that is fully compressible, and with no Doppler signal |  |  |  |  |  |  |
|  |  | Synovitis | An anechoic or hypoechoic intra-capsular area, different from cartilage with or without PD signal |  |  |  |  |  |  |
|  |  | Tenosynovitis | A hypoechoic rim around tendon with or without PD signal |  |  |  |  |  |  |
|  |  | PD activity | NR |  |  |  |  |  |  |
| Keen ^18^ | Hand:  1^st^ CMC  MCP 1-5  PIP 1-5  DIP 2-5 | Osteophytes | Cortical protrusions seen in two planes | No | NA | Dichotomous  0 = present  1 = absent  AND  0 = absent  1 = mild  2 = moderate  3 = Severe | LA 435 linear multifrequency transducer of 8-14 MHz. The B mode frequency used was 13 MHz. The power frequency was 10 MHz  The entire dorsal surface of the joint was imaged in the longitudinal plane | 15 experts in OA, US and outcome measures, met under the auspices of the Disease Characteristics in Hand OA Group | intrareader reliability  ĸ values of 0.087–1.0  Inter-reader reliability  ĸ values of 0.530 |
|  |  | Synovitis (synovial hypertrophy and effusion) | OMERACT definition of  Synovial hypertrophy and effusion developed for RA was applied ^7^ |  |  | Dichotomous  0 = present  1= absent  and  Semiquantitative  0 = no synovitis  1 = mild synovitis  2 = moderate synovitis  3 = Severe synovitis |  |  | intrareader reliability  ĸ values of 0.444–1.0  Inter-reader reliability  ĸ values of 0.398 |
|  |  | PD signal | A signal within a region  of grey scale synovitis |  |  | Dichotomous  0 = present  1 = absent  and  Semiquantitative  0 = no  1= mild  2 = moderate  3 = Severe |  |  | intrareader reliability  ĸ values of 0.211–1.0  Inter-reader reliability  ĸ values of 0.327 |
| Keen ^9^ | Hand:  1^st^ CMC  MCP 1-5  PIP 1-5  DIP 2-5 | Osteophytes | Cortical protrusion at the joint margin seen in two planes | No | NA | Present/absent ^18^ | Philips HDI 5000 SonoCT scanner. The joints were assessed with a 15-7 MHz hockey stick probe  Scanning across the longitudinal and transverse planes on the dorsal and palmar surfaces of the hand  ensuring the medial and lateral regions of the  joints were also visualised. The finger joints were held in a neutral position but extended and flexed as required to visualise pathology | Single ultrasonographer | The intra-reader reliability for the presence of osteophytosis  ĸ = 0.832 |
|  |  | Joint space narrowing | Documented as normal when the distances between superficial cortical surfaces of phalanges appeared normal |  |  | Normal or narrowed |  |  | The intra-reader reliability for US detected joint space narrowing  ĸ = 0.641 |
| Keen ^47^ | Hand:  1^st^ CMC  MCP 1-5  PIP 1-5  DIP 2-5 | Synovitis | A composite of synovial hypertrophy and effusion according to the OMERACT definition ^7^ | No | NA | Semiquantitative  0 = no synovitis  1 = mild synovitis  2 = moderate synovitis  3 = Severe synovitis ^11^ | Joints were assessed globally, scanning on both the dorsal and palmar aspects of the hand in both the longitudinal and transverse planes | Single ultrasonographer | Intrareader reliability  ĸw = 0.62 |
|  |  | PD signal | Areas of color signal within the joint capsule, when the gain was adjusted to exclude only background noise |  |  | Semiquantitative  0 = no  1 = mild  2 = moderate  3 = Severe ^11^ |  |  | Intrareader reliability  ĸw = 0.97 |
|  |  | Osteophytes | Cortical protrusions  seen in two planes |  |  | Scored by counting the number of osteophytes at each joint |  |  | Intrareader reliability was almost perfect ĸ = 0.83 |
|  |  | Joint space narrowing | The joint space was considered reduced when the space between the superficial cortical margins appeared reduced, or was assumed to be reduced because osteophytes prevented visualisation |  |  | A surrogate of radiographic joint space narrowing was used |  |  | Intrareader reliability was  substantial ĸ = 0.64 |

US, Ultrasound; USI, Ultrasound imaging; OA, Osteoarthritis; RA, Rheumatoid arthritis; MTPJ, metatarsophalangeal joint; PIP, Proximal interphalangeal joint; DIP, Distal interphalangeal joint; STJ, Subtalar joint; TNJ, Talonavicular joint; NCJ-M, navicular cuneiform joint medial; NCJ-I, navicular cuneiform joint intermediate; CMC, Carpometacarpal joint; MCP, metacarpophalangeal joint; MSK, Musculoskeletal; PD, power Doppler; κw, Weighted Kappa; κ, Kappa; ICC, Intraclass correlation coefficient; ACR, American College of Rheumatology; EULAR, European League Against Rheumatism; OMERACT, Outcome Measures in Rheumatology; NR, Not reported; NA, Not appliacable.

**References**

1. Zabotti A, Filippou G, Canzoni M, Adinolfi A, Picerno V, Carrara G, et al. OMERACT agreement and reliability study of ultrasonographic elementary lesions in osteoarthritis of the foot. RMD open 2019; 5: e000795.

2. Terslev L, Naredo E, Aegerter P, Wakefield RJ, Backhaus M, Balint P, et al. Scoring ultrasound synovitis in rheumatoid arthritis: a EULAR-OMERACT ultrasound taskforce-Part 2: reliability and application to multiple joints of a standardised consensus-based scoring system. RMD open 2017; 3: e000427.

3. D'Agostino MA, Boers M, Wakefield RJ, Hammer HB, Vittecoq O, Filippou G, et al. Exploring a new ultrasound score as a clinical predictive tool in patients with rheumatoid arthritis starting abatacept: results from the APPRAISE study. RMD open 2016; 2: e000237.

4. Mathiessen A, Haugen IK, Slatkowsky-Christensen B, Bøyesen P, Kvien TK, Hammer HB. Ultrasonographic assessment of osteophytes in 127 patients with hand osteoarthritis: exploring reliability and associations with MRI, radiographs and clinical joint findings. Annals of the rheumatic diseases 2013; 72: 51-56.

5. Iagnocco A, Conaghan P, Aegerter P, Möller I, Bruyn G, Chary-Valckenaere I, et al. The reliability of musculoskeletal ultrasound in the detection of cartilage abnormalities at the metacarpo-phalangeal joints. Osteoarthritis and cartilage 2012; 20: 1142-1146.

6. Iagnocco A, Filippucci E, Riente L, Meenagh G, Delle Sedie A, Sakellariou G, et al. Ultrasound imaging for the rheumatologist XXXV. Sonographic assessment of the foot in patients with osteoarthritis. 2011.

7. Wakefield RJ, Balint PV, Szkudlarek M, Filippucci E, Backhaus M, D'Agostino MA, et al. Musculoskeletal ultrasound including definitions for ultrasonographic pathology. The Journal of rheumatology 2005; 32: 2485-2487.

8. Backhaus M. Working group for musculoskeletal ultrasound in the EULAR standing Committee on International clinical studies including therapeutic trials. Guidelines for musculoskeletal ultrasound in rheumatology. Ann Rheum Dis 2001; 60: 641-649.

9. Keen HI, Wakefield RJ, Grainger AJ, Hensor EM, Emery P, Conaghan PG. Can ultrasonography improve on radiographic assessment in osteoarthritis of the hands? A comparison between radiographic and ultrasonographic detected pathology. Annals of the rheumatic diseases 2008; 67: 1116-1120.

10. Fjellstad CM, Mathiessen A, Slatkowsky-Christensen B, Kvien TK, Hammer HB, Haugen IK. Associations Between Ultrasound-Detected Synovitis, Pain, and Function in Interphalangeal and Thumb Base Osteoarthritis: Data From the Nor-Hand Cohort. Arthritis care & research 2020; 72: 1530-1535.

11. Keen HI, Lavie F, Wakefield RJ, D'Agostino MA, Hammer HB, Hensor E, et al. The development of a preliminary ultrasonographic scoring system for features of hand osteoarthritis. Annals of the Rheumatic Diseases 2008; 67: 651-655.

12. Steen Pettersen P, Neogi T, Magnusson K, Hammer HB, Uhlig T, Kvien TK, et al. Associations Between Radiographic and Ultrasound‐Detected Features in Hand Osteoarthritis and Local Pressure Pain Thresholds. Arthritis & rheumatology 2020; 72: 966-971.

13. Besselink NJ, Jacobs JWG, Westgeest AAA, van der Meijde P, Welsing PMJ, Marijnissen ACA, et al. Can optical spectral transmission assess ultrasound-detected synovitis in hand osteoarthritis? PLoS One 2019; 14: e0209761.

14. Besselink NJ, van der Meijde P, Rensen WH, Meijer PB, Marijnissen AC, van Laar JM, et al. Optical spectral transmission to assess inflammation in hand and wrist joints of rheumatoid arthritis patients. Rheumatology 2018; 57: 865-872.

15. Backhaus M, Burmester G, Gerber T, Grassi W, Machold K, Swen W, et al. Guidelines for musculoskeletal ultrasound in rheumatology. Annals of the rheumatic diseases 2001; 60: 641-649.

16. Oo WM, Deveza LA, Duong V, Fu K, Linklater JM, Riordan EA, et al. Musculoskeletal ultrasound in symptomatic thumb-base osteoarthritis: clinical, functional, radiological and muscle strength associations. BMC musculoskeletal disorders 2019; 20: 220.

17. Hammer HB, Bolton-King P, Bakkeheim V, Berg TH, Sundt E, Kongtorp AK, et al. Examination of intra and interrater reliability with a new ultrasonographic reference atlas for scoring of synovitis in patients with rheumatoid arthritis. Annals of the rheumatic diseases 2011; 70: 1995-1998.

18. Keen HI, Lavie F, Wakefield RJ, D'Agostino MA, Hammer HB, Hensor E, et al. The development of a preliminary ultrasonographic scoring system for features of hand osteoarthritis. Annals Of The Rheumatic Diseases 2008; 67: 651-655.

19. Kroon FPB, van Beest S, Ermurat S, Kortekaas MC, Bloem JL, Reijnierse M, et al. In thumb base osteoarthritis structural damage is more strongly associated with pain than synovitis. Osteoarthritis and cartilage 2018; 26: 1196-1202.

20. Kortekaas MC, Kwok WY, Reijnierse M, Watt I, Huizinga TW, Kloppenburg M, et al. Pain in hand osteoarthritis is associated with inflammation: the value of ultrasound. Annals of the rheumatic diseases 2010; 69: 1367-1369.

21. Sivakumaran P, Hussain S, Ciurtin C. Comparison between Several Ultrasound Hand Joint Scores and Conventional Radiography in Diagnosing Hand Osteoarthritis. Ultrasound in medicine & biology 2018; 44: 544-550.

22. Mandl P, Naredo E, Wakefield RJ, Conaghan PG, D’Agostino M. A systematic literature review analysis of ultrasound joint count and scoring systems to assess synovitis in rheumatoid arthritis according to the OMERACT filter. The Journal of rheumatology 2011; 38: 2055-2062.

23. Mandl P, Naredo E, Wakefield RJ, Conaghan PG, D’AGOSTINO MA. A systematic literature review analysis of ultrasound joint count and scoring systems to assess synovitis in rheumatoid arthritis according to the OMERACT filter. The Journal of rheumatology 2011; 38: 2055-2062.

24. Möller I, Bong D, Naredo E, Filippucci E, Carrasco I, Moragues C, et al. Ultrasound in the study and monitoring of osteoarthritis. Osteoarthritis and cartilage 2008; 16 Suppl 3: S4-S7.

25. Magnusson K, Mathiessen A, Hammer HB, Kvien TK, Slatkowsky-Christensen B, Natvig B, et al. Smoking and alcohol use are associated with structural and inflammatory hand osteoarthritis features. Scandinavian journal of rheumatology 2017; 46: 388-395.

26. Mathiessen A, Slatkowsky-Christensen B, Kvien TK, Haugen IK, Hammer HB. Ultrasound-detected osteophytes predict the development of radiographic and clinical features of hand osteoarthritis in the same finger joints 5 years later. RMD Open 2017; 3: e000505-e000505.

27. Mathiessen A, Slatkowsky-Christensen B, Kvien TK, Hammer HB, Haugen IK. Ultrasound-detected inflammation predicts radiographic progression in hand osteoarthritis after 5 years. Annals of the rheumatic diseases 2016; 75: 825-830.

28. Spolidoro Paschoal NdO, Natour J, Machado FS, Alcântara Veiga de Oliveira H, Vilar Furtado RN. Interphalangeal Joint Sonography of Symptomatic Hand Osteoarthritis: Clinical and Functional Correlation. Journal Of Ultrasound In Medicine: Official Journal Of The American Institute Of Ultrasound In Medicine 2017; 36: 311-319.

29. Szkudlarek M, Court‐Payen M, Jacobsen S, Klarlund M, Thomsen HS, Østergaard M. Interobserver agreement in ultrasonography of the finger and toe joints in rheumatoid arthritis. Arthritis & Rheumatism: Official Journal of the American College of Rheumatology 2003; 48: 955-962.

30. Hammer HB, Iagnocco A, Mathiessen A, Filippucci E, Gandjbakhch F, Kortekaas MC, et al. Global ultrasound assessment of structural lesions in osteoarthritis: a reliability study by the OMERACT ultrasonography group on scoring cartilage and osteophytes in finger joints. Annals Of The Rheumatic Diseases 2016; 75: 402-407.

31. Haugen IK, Mathiessen A, Slatkowsky-Christensen B, Magnusson K, Bøyesen P, Sesseng S, et al. Synovitis and radiographic progression in non-erosive and erosive hand osteoarthritis: is erosive hand osteoarthritis a separate inflammatory phenotype? Osteoarthritis and cartilage 2016; 24: 647-654.

32. Kortekaas MC, Kwok WY, Reijnierse M, Stijnen T, Kloppenburg M. Brief Report: Association of Inflammation With Development of Erosions in Patients With Hand Osteoarthritis: A Prospective Ultrasonography Study. Arthritis & Rheumatology 2016; 68: 392-397.

33. Kortekaas MC, Kwok W-Y, Reijnierse M, Kloppenburg M. Inflammatory ultrasound features show independent associations with progression of structural damage after over 2 years of follow-up in patients with hand osteoarthritis. Annals Of The Rheumatic Diseases 2015; 74: 1720-1724.

34. Kortekaas MC, Kwok W-Y, Reijnierse M, Watt I, Huizinga TW, Kloppenburg M. Pain in hand osteoarthritis is associated with inflammation: the value of ultrasound. Annals of the rheumatic diseases 2010; 69: 1367-1369.

35. Mancarella L, Addimanda O, Pelotti P, Pignotti E, Pulsatelli L, Meliconi R. Ultrasound detected inflammation is associated with the development of new bone erosions in hand osteoarthritis: a longitudinal study over 3.9 years. Osteoarthritis and cartilage 2015; 23: 1925-1932.

36. Abraham AM, Pearce MS, Mann KD, Francis RM, Birrell F. Population prevalence of ultrasound features of osteoarthritis in the hand, knee and hip at age 63 years: the Newcastle thousand families birth cohort. BMC musculoskeletal disorders 2014; 15: 162.

37. Keen HI, Conaghan PG. Ultrasonography in osteoarthritis. Radiologic clinics 2009; 47: 581-594.

38. Kortekaas MC, Kwok WY, Reijnierse M, Huizinga TW, Kloppenburg M. Follow-up study of inflammatory ultrasound features in hand osteoarthritis over a period of 3 months: variable as well as constant. Osteoarthritis and cartilage 2014; 22: 40-43.

39. Usón J, Fernández-Espartero C, Villaverde V, Condés E, Godo J, Martínez-Blasco MJ, et al. Symptomatic and asymptomatic interphalageal osteoarthritis: An ultrasonographic study. Reumatologia clinica 2014; 10: 278-282.

40. Kortekaas MC, Kwok WY, Reijnierse M, Huizinga TW, Kloppenburg M. In erosive hand osteoarthritis more inflammatory signs on ultrasound are found than in the rest of hand osteoarthritis. Annals of the rheumatic diseases 2013; 72: 930-934.

41. Vlychou M, Koutroumpas A, Alexiou I, Fezoulidis I, Sakkas LI. High-resolution ultrasonography and 3.0 T magnetic resonance imaging in erosive and nodal hand osteoarthritis: high frequency of erosions in nodal osteoarthritis. Clinical rheumatology 2013; 32: 755-762.

42. McNally E. Ultrasound of the small joints of the hands and feet: current status. Skeletal radiology 2008; 37: 99-113.

43. Vlychou M, Koutroumpas A, Malizos K, Sakkas LI, Vlychou M, Koutroumpas A, et al. Ultrasonographic evidence of inflammation is frequent in hands of patients with erosive osteoarthritis. Osteoarthritis and cartilage 2009; 17: 1283-1287.

44. Arrestier S, Rosenberg C, Etchepare F, Rozenberg S, Foltz V, Fautrel B, et al. Ultrasound features of nonstructural lesions of the proximal and distal interphalangeal joints of the hands in patients with finger osteoarthritis. Joint, bone, spine: revue du rhumatisme 2011; 78: 65-69.

45. Kortekaas MC, Kwok WY, Reijnierse M, Huizinga TW, Kloppenburg M, Kortekaas MC, et al. Osteophytes and joint space narrowing are independently associated with pain in finger joints in hand osteoarthritis. Annals of the rheumatic diseases 2011; 70: 1835-1837.

46. Mancarella L, Magnani M, Addimanda O, Pignotti E, Galletti S, Meliconi R. Ultrasound-detected synovitis with power Doppler signal is associated with severe radiographic damage and reduced cartilage thickness in hand osteoarthritis. Osteoarthritis and cartilage 2010; 18: 1263-1268.

47. Keen HI, Wakefield RJ, Grainger AJ, Hensor EM, Emery P, Conaghan PG. An ultrasonographic study of osteoarthritis of the hand: synovitis and its relationship to structural pathology and symptoms. Arthritis care & research 2008; 59: 1756-1763.
