# Supplementary material for: Evaluation of osteoarthritic features in peripheral joints by ultrasound imaging: A systematic review
Source: Osteoarthr Cartil Open. 2021 Jul 16;3(3):100194. doi: 10.1016/j.ocarto.2021.100194 (PMC9718269; doi:10.1016/j.ocarto.2021.100194)
Supplement: Multimedia component 3 [file mmc3.docx]

**Evaluation of osteoarthritic features in peripheral joints by ultrasound imaging: a systematic review**

**Supplementary Material 3: CASP checklist scores of included studies**

**CASP Checklist for Cohort studies**

|  | | **CASP criteria** | | | | | | | | | | | | | | | |
| --- | --- | --- | --- | --- | --- | --- | --- | --- | --- | --- | --- | --- | --- | --- | --- | --- | --- |
|  |  | 1 | 2 | 3 | 4 | 5 (a) | | 5 (b) | 6 (a) | 6 (b) | 7 | 8 | 9 | 10 | 11 | 12 | Quality Score |
| Abraham ^1^ | | Y | CT | CT | CT | N | | N | NA | NA | Y | Y | Y | Y | Y | Y | 7/14 |
| Besselink ^2^ | | Y | Y | CT | Y | Y | | Y | NA | NA | Y | Y | Y | CT | CT | CT | 8/14 |
| Fjellstat ^3^ | | Y | Y | Y | CT | Y | | Y | NA | NA | Y | Y | Y | Y | Y | Y | 11/14 |
| Hammer ^4^ | | Y | Y | Y | Y | N | | CT | NA | NA | Y | Y | Y | CT | Y | Y | 9/14 |
| Iagnocco ^5^ | | Y | Y | Y | Y | N | | CT | NA | NA | Y | CT | Y | CT | Y | Y | 8/14 |
| Iagnocco ^6^ | | Y | Y | CT | CT | N | | CT | NA | NA | Y | CT | Y | CT | CT | Y | 5/14 |
| Keen ^7^ | | Y | CT | CT | CT | N | | CT | NA | NA | Y | CT | Y | CT | CT | Y | 4/14 |
| Keen ^8^ | | Y | Y | Y | Y | N | | CT | NA | NA | Y | CT | Y | Y | Y | Y | 9/14 |
| Kortekaas ^9^ | | Y | Y | Y | Y | Y | | Y | NA | NA | Y | Y | Y | Y | Y | Y | 12/14 |
| Kortekaas ^10^ | | Y | Y | Y | Y | Y | | Y | Y | Y | Y | Y | Y | Y | Y | Y | 14/14 |
| Kortekaas ^11^ | | Y | Y | Y | Y | Y | | Y | NA | NA | Y | Y | Y | Y | Y | Y | 12/14 |
| Kortekaas ^12^ | | Y | Y | Y | Y | Y | | Y | Y | Y | Y | Y | Y | Y | Y | Y | 14/14 |
| Kortekaas ^13^ | | Y | Y | Y | Y | Y | | Y | Y | Y | Y | Y | Y | Y | Y | Y | 14/14 |
| Kortekaas ^14^ | | Y | Y | Y | Y | Y | | Y | NA | NA | Y | Y | Y | Y | Y | Y | 12/14 |
| Kroon ^15^ | | Y | Y | CT | Y | Y | | Y | NA | NA | Y | Y | Y | Y | Y | Y | 11/14 |
| Magnusson ^16^ | | Y | Y | CT | CT | Y | | Y | NA | NA | Y | Y | Y | Y | Y | CT | 10/14 |
| Mathiessen ^17^ | | Y | CT | Y | Y | N | | N | NA | NA | Y | CT | Y | Y | Y | Y | 8/14 |
| Mathiessen ^18^ | | Y | CT | CT | Y | Y | | Y | Y | Y | Y | Y | Y | Y | Y | Y | 12/14 |
| Mathiessen ^19^ | | Y | CT | CT | Y | Y | | Y | Y | Y | Y | Y | Y | Y | Y | Y | 12/14 |
| Oo ^20^ | | Y | CT | Y | Y | Y | | Y | NA | NA | Y | Y | Y | Y | Y | Y | 11/14 |
| Spolidoro ^21^ | | Y | CT | Y | CT | N | | N | Y | Y | Y | CT | Y | Y | Y | Y | 9/14 |
| Steen Pettersen ^22^ | | Y | CT | CT | CT | Y | | Y | NA | NA | Y | Y | Y | Y | Y | Y | 9/14 |
| Sivakumaran ^23^ | | Y | CT | Y | Y | N | | N | NA | NA | Y | CT | Y | Y | Y | Y | 8/14 |
| Uson ^24^ | | Y | CT | Y | Y | N | | N | NA | NA | Y | CT | Y | CT | Y | Y | 7/14 |
| Vlychou ^25^ | | Y | Y | Y | Y | N | | N | NA | NA | Y | CT | Y | CT | Y | Y | 8/14 |
| Zabotti ^26^ | | Y | CT | Y | Y | N | | N | NA | NA | Y | CT | Y | CT | CT | Y | 6/14 |
| 1 | Clearly focused issue stated | | | | | |  |  |  |  |  |  |  |  |  |  |  |
| 2 | Appropriate recruitment | | | | | |  |  |  |  |  |  |  |  |  |  |  |
| 3 | Exposure accurately measured to minimise bias | | | | | |  |  |  |  |  |  |  |  |  |  |  |
| 4 | Outcome accurately measured to minimise bias | | | | | |  |  |  |  |  |  |  |  |  |  |  |
| 5 (a) | Confounding factors identified | | | | | |  |  |  |  |  |  |  |  |  |  |  |
| 5 (b) | Confounding factors accounted | | | | | |  |  |  |  |  |  |  |  |  |  |  |
| 6 (a) | Subjects follow up is complete | | | | | |  |  |  |  |  |  |  |  |  |  |  |
| 6 (b) | Subjects follow-up is long enough | | | | | |  |  |  |  |  |  |  |  |  |  |  |
| 7 | Clear results | | | | | |  |  |  |  |  |  |  |  |  |  |  |
| 8 | Precise statistical results | | | | | |  |  |  |  |  |  |  |  |  |  |  |
| 9 | Results are believable | | | | | |  |  |  |  |  |  |  |  |  |  |  |
| 10 | Ability to generalise results to local population | | | | | |  |  |  |  |  |  |  |  |  |  |  |
| 11 | Interpretation related to the existing evidence | | | | | |  |  |  |  |  |  |  |  |  |  |  |
| 12 | Clear implications of this study for practice | | | | | |  |  |  |  |  |  |  |  |  |  |  |

Y: Yes; N: No; CT: Can’t Tell; NA: Not Applicable

**CASP Checklist for Case Control studies**

| **CASP criteria** | | | | | | | | | | | | | | | | |
| --- | --- | --- | --- | --- | --- | --- | --- | --- | --- | --- | --- | --- | --- | --- | --- | --- |
|  | | 1 | 2 | 3 | 4 | 5 | | 6 (a) | 6 (b) | 7 | 8 | 9 | 10 | | 11 | Quality score |
| Arrestier ^27^ | | Y | Y | Y | CT | CT | | Y | N | NA | NA | Y | Y | | CT | 6/12 |
| Haugen ^28^ | | Y | Y | Y | Y | Y | | CT | Y | NA | NA | Y | Y | | Y | 8/12 |
| Keen ^29^ | | Y | Y | Y | Y | Y | | Y | CT | NA | NA | Y | Y | | Y | 8/12 |
| Mancarella ^30^ | | Y | Y | Y | CT | Y | | Y | Y | NA | NA | Y | Y | | Y | 8/12 |
| Mancarella ^31^ | | Y | Y | Y | CT | Y | | Y | CT | NA | NA | Y | Y | | Y | 7/12 |
| Vlychou ^32^ | | Y | Y | Y | CT | Y | | CT | N | NA | NA | Y | Y | | CT | 5/12 |
| 1 | Clearly focused issue stated | | | | | |  |  |  |  |  |  |  |  |  |  |
| 2 | Appropriate method to answer question | | | | | |  |  |  |  |  |  |  |  |  |  |
| 3 | Cases recruited in an acceptable way | | | | | |  |  |  |  |  |  |  |  |  |  |
| 4 | Controls recruited in an acceptable way | | | | | |  |  |  |  |  |  |  |  |  |  |
| 5 (a) | Exposure accurately measured to minimise bias | | | | | |  |  |  |  |  |  |  |  |  |  |
| 5 (b) | Groups treated equally | | | | | |  |  |  |  |  |  |  |  |  |  |
| 6 (a) | Account of the potential confounding factors in the design and/or in their analysis | | | | | | | | | | | | |  |  |  |
| 6 (b) | How large was the treatment effect? | | | | | |  |  |  |  |  |  |  |  |  |  |
| 7 | Precise estimate of the treatment effect | | | | | |  |  |  |  |  |  |  |  |  |  |
| 8 | Results are believable | | | | | |  |  |  |  |  |  |  |  |  |  |
| 9 | Ability to generalise results to local population | | | | | |  |  |  |  |  |  |  |  |  |  |
| 10 | Interpretation related to the existing evidence | | | | | |  |  |  |  |  |  |  |  |  |  |

**References**

1. Abraham AM, Pearce MS, Mann KD, Francis RM, Birrell F. Population prevalence of ultrasound features of osteoarthritis in the hand, knee and hip at age 63 years: the Newcastle thousand families birth cohort. BMC musculoskeletal disorders 2014; 15: 162.

2. Besselink NJ, Jacobs JWG, Westgeest AAA, van der Meijde P, Welsing PMJ, Marijnissen ACA, et al. Can optical spectral transmission assess ultrasound-detected synovitis in hand osteoarthritis? PLoS One 2019; 14: e0209761.

3. Fjellstad CM, Mathiessen A, Slatkowsky-Christensen B, Kvien TK, Hammer HB, Haugen IK. Associations Between Ultrasound-Detected Synovitis, Pain, and Function in Interphalangeal and Thumb Base Osteoarthritis: Data From the Nor-Hand Cohort. Arthritis care & research 2020; 72: 1530-1535.

4. Hammer HB, Iagnocco A, Mathiessen A, Filippucci E, Gandjbakhch F, Kortekaas MC, et al. Global ultrasound assessment of structural lesions in osteoarthritis: a reliability study by the OMERACT ultrasonography group on scoring cartilage and osteophytes in finger joints. Annals of the rheumatic diseases 2016; 75: 402-407.

5. Iagnocco A, Conaghan P, Aegerter P, Möller I, Bruyn G, Chary-Valckenaere I, et al. The reliability of musculoskeletal ultrasound in the detection of cartilage abnormalities at the metacarpo-phalangeal joints. Osteoarthritis and cartilage 2012; 20: 1142-1146.

6. Iagnocco A, Filippucci E, Riente L, Meenagh G, Delle Sedie A, Sakellariou G, et al. Ultrasound imaging for the rheumatologist XXXV. Sonographic assessment of the foot in patients with osteoarthritis. Clinical and experimental rheumatology 2011; 29: 757-762.

7. Keen HI, Lavie F, Wakefield RJ, D'Agostino MA, Hammer HB, Hensor E, et al. The development of a preliminary ultrasonographic scoring system for features of hand osteoarthritis. Annals of the rheumatic diseases 2008; 67: 651-655.

8. Keen HI, Wakefield RJ, Grainger AJ, Hensor EM, Emery P, Conaghan PG. An ultrasonographic study of osteoarthritis of the hand: synovitis and its relationship to structural pathology and symptoms. Arthritis care & research 2008; 59: 1756-1763.

9. Kortekaas MC, Kwok WY, Reijnierse M, Huizinga TW, Kloppenburg M. In erosive hand osteoarthritis more inflammatory signs on ultrasound are found than in the rest of hand osteoarthritis. Annals of the rheumatic diseases 2013; 72: 930-934.

10. Kortekaas MC, Kwok WY, Reijnierse M, Huizinga TW, Kloppenburg M. Follow-up study of inflammatory ultrasound features in hand osteoarthritis over a period of 3 months: variable as well as constant. Osteoarthritis and cartilage 2014; 22: 40-43.

11. Kortekaas MC, Kwok WY, Reijnierse M, Huizinga TW, Kloppenburg M, Kortekaas MC, et al. Osteophytes and joint space narrowing are independently associated with pain in finger joints in hand osteoarthritis. Annals of the rheumatic diseases 2011; 70: 1835-1837.

12. Kortekaas MC, Kwok WY, Reijnierse M, Kloppenburg M. Inflammatory ultrasound features show independent associations with progression of structural damage after over 2 years of follow-up in patients with hand osteoarthritis. Annals of the rheumatic diseases 2015; 74: 1720-1724.

13. Kortekaas MC, Kwok WY, Reijnierse M, Stijnen T, Kloppenburg M. Brief Report: Association of Inflammation With Development of Erosions in Patients With Hand Osteoarthritis: A Prospective Ultrasonography Study. Arthritis care & research 2016; 68: 392-397.

14. Kortekaas MC, Kwok WY, Reijnierse M, Watt I, Huizinga TW, Kloppenburg M, et al. Pain in hand osteoarthritis is associated with inflammation: the value of ultrasound. Annals of the rheumatic diseases 2010; 69: 1367-1369.

15. Kroon FPB, van Beest S, Ermurat S, Kortekaas MC, Bloem JL, Reijnierse M, et al. In thumb base osteoarthritis structural damage is more strongly associated with pain than synovitis. Osteoarthritis and cartilage 2018; 26: 1196-1202.

16. Magnusson K, Mathiessen A, Hammer HB, Kvien TK, Slatkowsky-Christensen B, Natvig B, et al. Smoking and alcohol use are associated with structural and inflammatory hand osteoarthritis features. Scandinavian journal of rheumatology 2017; 46: 388-395.

17. Mathiessen A, Haugen IK, Slatkowsky-Christensen B, Boyesen P, Kvien TK, Hammer HB. Ultrasonographic assessment of osteophytes in 127 patients with hand osteoarthritis: exploring reliability and associations with MRI, radiographs and clinical joint findings. Annals of the rheumatic diseases 2013; 72: 51-56.

18. Mathiessen A, Slatkowsky-Christensen B, Kvien TK, Hammer HB, Haugen IK. Ultrasound-detected inflammation predicts radiographic progression in hand osteoarthritis after 5 years. Annals of the rheumatic diseases 2016; 75: 825-830.

19. Mathiessen A, Slatkowsky-Christensen B, Kvien TK, Haugen IK, Berner Hammer H. Ultrasound-detected osteophytes predict the development of radiographic and clinical features of hand osteoarthritis in the same finger joints 5 years later. RMD open 2017; 3: e000505.

20. Oo WM, Deveza LA, Duong V, Fu K, Linklater JM, Riordan EA, et al. Musculoskeletal ultrasound in symptomatic thumb-base osteoarthritis: clinical, functional, radiological and muscle strength associations. BMC musculoskeletal disorders 2019; 20: 220.

21. Spolidoro Paschoal NdO, Natour J, Machado FS, Alcântara Veiga de Oliveira H, Vilar Furtado RN. Interphalangeal Joint Sonography of Symptomatic Hand Osteoarthritis: Clinical and Functional Correlation. Journal of ultrasound in medicine : official journal of the American Institute of Ultrasound in Medicine 2017; 36: 311-319.

22. Steen Pettersen P, Neogi T, Magnusson K, Hammer HB, Uhlig T, Kvien TK, et al. Associations Between Radiographic and Ultrasound‐Detected Features in Hand Osteoarthritis and Local Pressure Pain Thresholds. Arthritis & rheumatology 2020; 72: 966-971.

23. Sivakumaran P, Hussain S, Ciurtin C. Comparison between Several Ultrasound Hand Joint Scores and Conventional Radiography in Diagnosing Hand Osteoarthritis. Ultrasound in medicine & biology 2018; 44: 544-550.

24. Uson J, Fernandez-Espartero C, Villaverde V, Condes E, Godo J, Martinez-Blasco MJ, et al. Symptomatic and asymptomatic interphalageal osteoarthritis: An ultrasonographic study. Reumatologia clinica 2014; 10: 278-282.

25. Vlychou M, Koutroumpas A, Malizos K, Sakkas LI. Ultrasonographic evidence of inflammation is frequent in hands of patients with erosive osteoarthritis. Osteoarthritis and cartilage 2009; 17: 1283-1287.

26. Zabotti A, Filippou G, Canzoni M, Adinolfi A, Picerno V, Carrara G, et al. OMERACT agreement and reliability study of ultrasonographic elementary lesions in osteoarthritis of the foot. RMD open 2019; 5: e000795.

27. Arrestier S, Rosenberg C, Etchepare F, Rozenberg S, Foltz V, Fautrel B, et al. Ultrasound features of nonstructural lesions of the proximal and distal interphalangeal joints of the hands in patients with finger osteoarthritis. Joint bone spine 2011; 78: 65-69.

28. Haugen IK, Mathiessen A, Slatkowsky-Christensen B, Magnusson K, Bøyesen P, Sesseng S, et al. Synovitis and radiographic progression in non-erosive and erosive hand osteoarthritis: is erosive hand osteoarthritis a separate inflammatory phenotype? Osteoarthritis and cartilage 2016; 24: 647-654.

29. Keen HI, Wakefield RJ, Grainger AJ, Hensor EMA, Emery P, Conaghan PG. Can ultrasonography improve on radiographic assessment in osteoarthritis of the hands? A comparison between radiographic and ultrasonographic detected pathology. Annals of the rheumatic diseases 2008; 67: 1116-1120.

30. Mancarella L, Addimanda O, Pelotti P, Pignotti E, Pulsatelli L, Meliconi R. Ultrasound detected inflammation is associated with the development of new bone erosions in hand osteoarthritis: a longitudinal study over 3.9 years. Osteoarthritis and cartilage 2015; 23: 1925-1932.

31. Mancarella L, Magnani M, Addimanda O, Pignotti E, Galletti S, Meliconi R. Ultrasound-detected synovitis with power Doppler signal is associated with severe radiographic damage and reduced cartilage thickness in hand osteoarthritis. Osteoarthritis and cartilage 2010; 18: 1263-1268.

32. Vlychou M, Koutroumpas A, Alexiou I, Fezoulidis I, Sakkas LI. High-resolution ultrasonography and 3.0 T magnetic resonance imaging in erosive and nodal hand osteoarthritis: high frequency of erosions in nodal osteoarthritis. Clinical rheumatology 2013; 32: 755-762.
