# Supplementary material for: Evaluation of osteoarthritic features in peripheral joints by ultrasound imaging: A systematic review
Source: Osteoarthr Cartil Open. 2021 Jul 16;3(3):100194. doi: 10.1016/j.ocarto.2021.100194 (PMC9718269; doi:10.1016/j.ocarto.2021.100194)
Supplement: Multimedia component 4 [file mmc4.docx]

**Evaluation of osteoarthritic features in peripheral joints by ultrasound imaging: a systematic review**

**Supplementary Material 4: Reliability of grading of OA features using USI**

|  |  | **Intra-rater reliability** | | | **Inter-rater reliability** | | |  |
| --- | --- | --- | --- | --- | --- | --- | --- | --- |
| **USI feature** | **Study** | **Semiquantitative** | **Dichotomous (present or absent)** | **Continuous (mm)** | **Semiquantitative** | **Dichotomous (present or absent)** | **Continuous (mm)** | **Sonographer** |
| **Synovial hypertrophy** | Zabotti ^1^ | ĸ = 0.48 | ĸ = 0.64 | NR | ĸ = 0.63 and 0.59 | ĸ = 0.50 and 0.64 | NR | Eleven rheumatologists all experienced in US and members of the OMERACT group |
|  | Mancarella ^2^ | NR | ĸ = 0.84 | NR | NR | NR | NR | Two experienced MSK sonographers |
|  | Spolidoro, ^3^ | Dorsal  ĸ = 0.67  Palmer  ĸ = 0.44 | NR | NR | Dorsal  ĸ = 0.62  Palmer  ĸ = 0.50 | NR | NR | Two experienced rheumatologists in MSK USI |
|  | Korteĸaas ^4^ | ICC = 0.84 | NR | NR | NR | NR | NR | One experienced ultrasonographer, scoring together in consensus with a second ultrasonographer. Both blinded to clinical findings |
|  | Korteĸaas ^5^ | ICC = 0.73 | NR | NR | NR | NR | NR | US assessment by one ultrasonographer and scored together with a second ultrasonographer. |
|  | Korteĸaas ^6^ | ICC = 0.73 | NR | NR | NR | NR | NR | Two ultrasonographers blinded to clinical findings and PR scores |
|  | Korteĸaas ^7^ | ICC = 0.73 | NR | NR | NR | NR | NR | Two ultrasonographers |
| **Power Doppler signal** | Fjellstad ^8^ | NR | NR | NR | ĸ = 0.85 and ĸ = 0.92 | NR | NR | A trained medical student performed the US examination. Scoring was performed in consensus with an experienced ultrasonographer |
|  | Zabotti ^1^ | ĸ = 0.90 | NR | NR | ĸ= 0.87 and 0.89 | NR | NR | Eleven rheumatologists all experienced in US and members of the OMERACT group |
|  | Mancarella ^2^ | NR | ĸ = 0.78 | NR | NR | NR | NR | Two experienced MSK sonographers |
|  | Korteĸaas ^4^ | ICC = 0.62 | NR | NR | NR | NR | NR | One experienced ultrasonographer, scoring together in consensus with a second ultrasonographer. Both blinded to clinical findings |
|  | Korteĸaas ^5^ | ICC = 0.57 | NR | NR | NR | NR | NR | US assessment by one ultrasonographer and scored together with a second ultrasonographer |
|  | Korteĸaas ^6^ | ICC = 0.57 | NR | NR | NR | NR | NR | Two ultrasonographers blinded to clinical findings and PR scores |
|  | Korteĸaas ^7^ | ICC = 0.57 | NR | NR | NR | NR | NR | Two ultrasonographers |
|  | Oo ^9^ | NR | ĸ = 0.89 | NR | NR | NR | NR | Sonographer, experience in MSK US |
|  | Steen ^10^ | NR | NR | NR | ĸ = 0.79 | NR | NR | A trained medical student performed the US examinations. Initial scorings were done in consensus with an experienced ultrasonographer |
|  | Keen ^11^ | ĸ = 0.09-1.0 | ĸ = 0.21-1.0 | NR | ĸ = 0.23 | ĸ = 0.33 | NR | 15 experts in OA, US and outcome measures, met under the auspices of the Disease Characteristics in Hand OA Group. Reliability exercise involved seven examiners |
|  | Keen ^12^ | ĸ=0.97 | NR | NR | NR | NR | NR | Single ultrasonographer |
|  | Mathiessen ^13^ | NR | NR | NR | ĸ=0.93 | NR | NR | One trainee and one experienced rheumatologist performed the US assessments together and reached consensus on each scoring |
|  | Vlychou ^14^ | NR | ĸ = 0.81 | NR | NR | NR | NR | Trained radiologist with a 4-year experience in MSK US. Blinded to radiographic and clinical data |
|  | Mancarella ^15^ | NR | ĸ = 0.86 | NR | NR | NR | NR | Single sonographer experienced in MSK US, blinded to PR data |
| **Synovitis** | Fjellstad ^8^ | NR | NR | NR | ĸ = 0.80 and ĸ = 0.92 | NR | NR | A trained medical student performed the US examination. Scoring was performed in consensus with an experienced ultrasonographer. |
|  | Mancarella ^2^ | NR | ĸ = 0.90 | NR | NR | NR | NR | Two experienced MSK sonographers |
|  | Steen ^10^ | NR | NR | NR | ĸ = 0.80 | NR | NR | A trained medical  student performed the US examinations. Initial scorings were done in consensus with an experienced ultrasonographer |
|  | Oo ^9^ | ĸ = 0.77 | NR | NR | NR | NR | NR | Sonographer, experience in MSK US |
|  | Mathiessen ^13^ | ĸ = 0.86 | NR | NR | ĸ = 0.74 | NR | NR | One trainee and one experienced rheumatologist  performed the US assessments together and reached consensus on each scoring |
|  | Keen ^12^ | ĸ = 0.62 | NR | NR | NR | NR | NR | Single ultrasonographer |
|  | Vlychou ^14^ | NR | ĸ =0.8 | NR | NR | NR | NR | Trained radiologist with a 4-year experience in MSK US. Blinded to radiographic and clinical data |
|  | Keen ^11^ | ĸ = 0.17-1.0 | ĸ = 0.07- 1.0 | NR | ĸ = 0.25 | ĸ = 0.40 | NR | 15 experts in OA, US and outcome measures, met under the auspices of the Disease Characteristics in Hand OA Group. Reliability exercise involved seven examiners |
|  | Vlychou ^16^ | NR | Agreement between USI and MRI  ĸ = 0.82 | NR | NR | NR | NR | Radiologist experienced in MSK US |
| **Joint effusion** | Zabotti ^1^ | NR | ĸ =0.67 | NR | NR | ĸ = 0.80 and 0.61 | NR | Eleven rheumatologists all experienced in US and members of the OMERACT group |
|  | Mancarella ^2^ | NR | ĸ = 0.83 | NR | NR | NR | NR | Two experienced MSK sonographers |
|  | Korteĸaas ^4^ | ICC = 0.84 | NR | NR | NR | NR | NR | One experienced ultrasonographer, scoring together in consensus with a second ultrasonographer. Both blinded to clinical findings |
|  | Korteĸaas ^5^ | ICC = 0.73 | NR | NR | NR | NR | NR | US assessment by one ultrasonographer and scored together with a second ultrasonographer |
|  | Korteĸaas ^6^ | ICC = 0.73 | NR | NR | NR | NR | NR | Two ultrasonographers blinded to clinical findings and PR scores |
|  | Korteĸaas ^7^ | ICC = 0.73 | NR | NR | NR | NR | NR | Two ultrasonographers |
|  | Vlychou ^14^ | NR | ĸ = 0.81 | NR | NR | NR | NR | Trained radiologist with a 4-year experience in MSK US. Blinded to radiographic and clinical data |
|  | Vlychou ^16^ | NR | Agreement between USI and MRI  ĸ = 0.87 | NR | NR | NR | NR | Radiologist experienced in MSK US |
|  | Mancarella ^15^ | NR | ĸ = 0.94 | NR | NR | NR | NR | Single sonographer experienced in MSK US, blinded to PR data |
| **Osteophytes** | Fjellstad ^8^ | NR | NR | NR | ĸ = 0.72 and ĸ = 0.80 | NR | NR | A trained medical student performed the US examination. Scoring was performed in consensus with an experienced ultrasonographer |
|  | Zabotti ^1^ | NR | ĸ=0.63 | NR | NR | ĸ = 0.54 and 0.58 | NR | Eleven rheumatologists all experienced in US and members of the OMERACT group |
|  | Abraham ^17^ | NR | NR | NR | NR | ĸ = 0.50- 0.69 | NR | Trained MSK Ultrasonographers |
|  | Oo ^9^ | ĸ = 0.79 | NR | NR | NR | NR | NR | Sonographer, experience in MSK US |
|  | Hammer ^18^ | ĸ = 0.69-0.89 | NR | NR | ĸ = 0.65-0.67 | NR | NR | 10 sonographers (9 were rheumatologists, experts in MSK USI and members of the OMERACT US group and one trainee fellow in rheumatology, highly experienced and had participated in the development of the USI atlas |
|  | Vlychou ^14^ | NR | ĸ = 0.81 | NR | NR | NR | NR | Trained radiologist with a 4-year experience in MSK US. Blinded to radiographic and clinical data |
|  | Vlychou ^16^ | NR | Agreement between USI and MRI  ĸ = 0.79 | NR | NR | NR | NR | Radiologist experienced in MSK US |
|  | Keen ^11^ | ĸ = 0.17-0.91 | ĸ= 0.09-1.0 | NR | ĸ = 0.38 | ĸ = 0.53 | NR | 15 experts in OA, US and outcome measures, met under the auspices of the Disease Characteristics in Hand OA Group. Reliability exercise involved seven examiners |
|  | Keen ^19^ | NR | NR | NR | NR | ĸ = 0.83 | NR | Single ultrasonographer |
|  | Keen ^12^ | NR | NR | NR | NR | ĸ = 0.83 | NR | Single ultrasonographer |
|  | Mathiessen ^20^ | ĸ = 0.91 | NR | NR | ĸ = 0.91 | NR | NR | One trainee and one experienced sonographer performed the ultrasound assessments together and reached consensus on each scoring |
|  | Korteĸaas ^5^ | ICC = 0.71 | NR | NR | NR | NR | NR | US assessment by one ultrasonographer and scored together with a second ultrasonographer |
|  | Korteĸaas ^6^ | ICC = 0.71 | NR | NR | NR | NR | NR | Two ultrasonographers blinded to clinical findings and PR scores. |
| **Cartilage damage** | Zabotti ^1^ | NR | ĸ = 0.64 | NR | NR | ĸ = 0.60 | NR | Eleven rheumatologists all experienced in US and members of the OMERACT group |
|  | Iagnocco ^21^ | NR | ĸ = 0.5-1.0 | NR | NR | ĸ = 0.39 - 0.80 | NR | Nine expert MSK ultrasonographers |
|  | Hammer ^18^ | ĸ = 0.46-0.66 | NR | NR | ĸ = 0.33- 0.39 | NR | NR | 10 sonographers (9 were rheumatologists, experts in MSK USI and members of the OMERACT US group and one trainee fellow in rheumatology, highly experienced and had participated in the development of the USI atlas |
|  | Mancarella ^15^ | NR | NR | ICC = 0.93 | NR | NR | NR | Single sonographer experienced in MSK US, blinded to PR data. |
| **Erosions** | Mancarella ^2^ | NR | ĸ = 0.87 | NR | NR | NR | NR | Two experienced MSK sonographers |
|  | Vlychou ^16^ | NR | Agreement between USI and MRI  ĸ = 0.84 | NR | NR | NR | NR | Radiologist experienced in MSK US |
|  | Vlychou ^14^ | NR | ĸ = 0.81 | NR | NR | NR | NR | Trained radiologist with a 4-year experience in MSK US. Blinded to radiographic and clinical data |
| **Joint space narrowing** | Keen ^19^ | NR | ĸ = 0.64 | NR | NR | NR | NR | Single ultrasonographer |
|  | Keen ^12^ | NR | ĸ = 0.64 | NR | NR | NR | NR | Single ultrasonographer |
| **Tenosynovitis** | Vlychou ^16^ | NR | Agreement between USI and MRI  ĸ = 0.83 | NR | NR | NR | NR | Radiologist experienced in MSK US |
|  | Vlychou ^14^ | NR | ĸ = 0.81 | NR | NR | NR | NR | Trained radiologist with a 4-year experience in MSK US. Blinded to radiographic and clinical data. |

NR, Not reported; ĸ, Kappa; ICC, Intraclass correlation coefficient; US, Ultrasound; USI, Ultrasound imaging; MRI, Magnetic resonance imaging; MSK, Musculoskeletal; OMERACT, Outcome Measures in Rheumatology.

**References**

1. Zabotti A, Filippou G, Canzoni M, Adinolfi A, Picerno V, Carrara G, et al. OMERACT agreement and reliability study of ultrasonographic elementary lesions in osteoarthritis of the foot. RMD open 2019; 5: e000795.

2. Mancarella L, Addimanda O, Pelotti P, Pignotti E, Pulsatelli L, Meliconi R. Ultrasound detected inflammation is associated with the development of new bone erosions in hand osteoarthritis: a longitudinal study over 3.9 years. Osteoarthritis & Cartilage 2015; 23: 1925-1932.

3. Spolidoro Paschoal NdO, Natour J, Machado FS, Alcântara Veiga de Oliveira H, Vilar Furtado RN. Interphalangeal Joint Sonography of Symptomatic Hand Osteoarthritis: Clinical and Functional Correlation. Journal of ultrasound in medicine 2017; 36: 311-319.

4. Kortekaas MC, Kwok WY, Reijnierse M, Kloppenburg M. Inflammatory ultrasound features show independent associations with progression of structural damage after over 2 years of follow-up in patients with hand osteoarthritis. Annals of the rheumatic diseases 2015; 74: 1720-1724.

5. Kortekaas MC, Kwok WY, Reijnierse M, Huizinga TW, Kloppenburg M. In erosive hand osteoarthritis more inflammatory signs on ultrasound are found than in the rest of hand osteoarthritis. Annals of the rheumatic diseases 2013; 72: 930-934.

6. Kortekaas MC, Kwok WY, Reijnierse M, Huizinga TW, Kloppenburg M, Kortekaas MC, et al. Osteophytes and joint space narrowing are independently associated with pain in finger joints in hand osteoarthritis. Annals of the rheumatic diseases 2011; 70: 1835-1837.

7. Kortekaas MC, Kwok WY, Reijnierse M, Watt I, Huizinga TW, Kloppenburg M, et al. Pain in hand osteoarthritis is associated with inflammation: the value of ultrasound. Annals of the rheumatic diseases 2010; 69: 1367-1369.

8. Fjellstad CM, Mathiessen A, Slatkowsky-Christensen B, Kvien TK, Hammer HB, Haugen IK. Associations Between Ultrasound-Detected Synovitis, Pain, and Function in Interphalangeal and Thumb Base Osteoarthritis: Data From the Nor-Hand Cohort. Arthritis care & research 2020; 72: 1530-1535.

9. Oo WM, Deveza LA, Duong V, Fu K, Linklater JM, Riordan EA, et al. Musculoskeletal ultrasound in symptomatic thumb-base osteoarthritis: clinical, functional, radiological and muscle strength associations. BMC Musculoskeletal Disorders 2019; 20: 1-9.

10. Steen Pettersen P, Neogi T, Magnusson K, Hammer HB, Uhlig T, Kvien TK, et al. Associations Between Radiographic and Ultrasound‐Detected Features in Hand Osteoarthritis and Local Pressure Pain Thresholds. Arthritis & rheumatology 2020; 72: 966-971.

11. Keen HI, Lavie F, Wakefield RJ, D'Agostino MA, Hammer HB, Hensor E, et al. The development of a preliminary ultrasonographic scoring system for features of hand osteoarthritis. Annals of the Rheumatic Diseases 2008; 67: 651-655.

12. Keen HI, Wakefield RJ, Grainger AJ, Hensor EM, Emery P, Conaghan PG. Can ultrasonography improve on radiographic assessment in osteoarthritis of the hands? A comparison between radiographic and ultrasonographic detected pathology. Annals of the rheumatic diseases 2008; 67: 1116-1120.

13. Mathiessen A, Slatkowsky-Christensen B, Kvien TK, Hammer HB, Haugen IK. Ultrasound-detected inflammation predicts radiographic progression in hand osteoarthritis after 5 years. Annals of the rheumatic diseases 2016; 75: 825-830.

14. Vlychou M, Koutroumpas A, Malizos K, Sakkas LI. Ultrasonographic evidence of inflammation is frequent in hands of patients with erosive osteoarthritis. Osteoarthritis & Cartilage 2009; 17: 1283-1287.

15. Mancarella L, Magnani M, Addimanda O, Pignotti E, Galletti S, Meliconi R. Ultrasound-detected synovitis with power Doppler signal is associated with severe radiographic damage and reduced cartilage thickness in hand osteoarthritis. Osteoarthritis & Cartilage 2010; 18: 1263-1268.

16. Vlychou M, Koutroumpas A, Alexiou I, Fezoulidis I, Sakkas LI. High-resolution ultrasonography and 3.0 T magnetic resonance imaging in erosive and nodal hand osteoarthritis: high frequency of erosions in nodal osteoarthritis. Clinical Rheumatology 2013; 32: 755-762.

17. Abraham AM, Pearce MS, Mann KD, Francis RM, Birrell F. Population prevalence of ultrasound features of osteoarthritis in the hand, knee and hip at age 63 years: the Newcastle thousand families birth cohort. BMC Musculoskeletal Disorders 2014; 15: 162.

18. Hammer HB, Iagnocco A, Mathiessen A, Filippucci E, Gandjbakhch F, Kortekaas MC, et al. Global ultrasound assessment of structural lesions in osteoarthritis: a reliability study by the OMERACT ultrasonography group on scoring cartilage and osteophytes in finger joints. Annals of the rheumatic diseases 2016; 75: 402-407.

19. Keen HI, Wakefield RJ, Grainger AJ, Hensor EM, Emery P, Conaghan PG. An ultrasonographic study of osteoarthritis of the hand: synovitis and its relationship to structural pathology and symptoms. Arthritis & Rheumatism 2008; 59: 1756-1763.

20. Mathiessen A, Haugen IK, Slatkowsky-Christensen B, Boyesen P, Kvien TK, Hammer HB. Ultrasonographic assessment of osteophytes in 127 patients with hand osteoarthritis: exploring reliability and associations with MRI, radiographs and clinical joint findings. Annals of the rheumatic diseases 2013; 72: 51-56.

21. Iagnocco A, Conaghan P, Aegerter P, Möller I, Bruyn G, Chary-Valckenaere I, et al. The reliability of musculoskeletal ultrasound in the detection of cartilage abnormalities at the metacarpo-phalangeal joints. Osteoarthritis and cartilage 2012; 20: 1142-1146.
